# Removing the association of random gene sets and survival time in cancers with positive random bias using fixed-point gene set

**DOI:** 10.1038/s41598-023-35588-5

**Published:** 2023-05-29

**Authors:** Maryam Maghsoudi, Rosa Aghdam, Changiz Eslahchi

**Affiliations:** 1 School of Biological Sciences, Institute for Research in Fundamental Sciences (IPM), Tehran, Iran; 2 Wisconsin Institute for Discovery, University of Wisconsin-Madison, Madison, WI 53715 USA; 3 Department of Computer and Data Sciences, Faculty of Mathematical Sciences, Shahid Beheshti University, Tehran, Iran

**Keywords:** Computational biology and bioinformatics, Systems biology, Biomarkers, Diseases

## Abstract

Cancer research aims to identify genes that cause or control disease progression. Although a wide range of gene sets have been published, they are usually in poor agreement with one another. Furthermore, recent findings from a gene-expression cohort of different cancer types, known as positive random bias, showed that sets of genes chosen randomly are significantly associated with survival time much higher than expected. In this study, we propose a method based on Brouwer’s fixed-point theorem that employs significantly survival-associated random gene sets and reveals a small fixed-point gene set for cancers with a positive random bias property. These sets significantly correspond to cancer-related pathways with biological relevance for the progression and metastasis of the cancer types they represent. Our findings show that our proposed significant gene sets are biologically related to each cancer type available in the cancer genome atlas with the positive random bias property, and by using these sets, positive random bias is significantly more reduced in comparison with state-of-the-art methods in this field. The random bias property is removed in 8 of these 17 cancer types, and the number of random sets of genes associated with survival time is significantly reduced in the remaining 9 cancers.

## Introduction

According to the American Cancer Society, cancer is the second most common cause of death in the US^1^. In addition, cancers are heterogeneous diseases, with comparable diagnoses and identical treatment regimens resulting in vastly different outcomes for patients. On the other hand, early cancer diagnosis and prognosis have substantial effects on patients’ therapeutic targets^2^. This has prompted researchers to seek out factors that can aid in predicting the course of cancer disease. The findings of numerous studies that relied solely on clinical characteristics such as lymph node status and histological grade to classify clinical outcomes demonstrated that these characteristics were insufficient. This has led to the development of studies considering genomic data (e.g., gene expression) alongside clinical features. In general, the goal of such studies was to select a preferably small number of genes, known as the signature, and to utilize them in predicting a patient’s survival outcome using gene expression profiles^3^.

Nonetheless, detecting a robust gene set across various datasets that can accurately predict a patient’s survival outcome has become a key challenge in cancer research. In the last two decades, numerous articles have been published on finding survival-relative genes in various cancer types, each proposing a gene set that was highly associated with cancer progression and metastasis^4–10^. Nevertheless, there was little overlap between the resulting gene sets from studies with different cohorts but similar analytical approaches^11^. Therefore, the lack of similarities between the reported gene sets in these studies indicates that the results depend on the cohorts being studied. As a result, identifying a robust gene set across multiple datasets that accurately predicts a patient’s outcome has become a formidable challenge in cancer research. In this regard, considering the cancer patient’s survival time is one of the most critical aspects of finding such a gene set^11^.

In 2012, Venet et al.^12^ argued this point and conducted a study to estimate the association between randomly selected gene sets and breast cancer patient survival time in a Netherlands Cancer Institute (NKI) cohort. As one might expect, using the expression of random genes to divide samples into two distinct groups results in groups that are not significantly different in terms of survival time, and samples are assigned to each group randomly. In other words, the *p*-values obtained from statistical tests comparing survival curves of the groups generated by random gene sets must be distributed normally, with only 5% of the *p*-values falling below 0.05^13^. By contrast, Venet’s analysis revealed that in the case of breast cancer, groups generated by many of the random gene sets showed a statistically significant difference in survival time. That is to say, these random gene sets were significantly associated with the patient’s survival time. Additionally, in some cases, the random gene sets were more significantly associated with survival time than some of the published signatures^12^. These findings suggest that many of the signatures identified through breast cancer gene expression analysis may not be causal to cancer progression, despite being significantly associated with survival time^13^. Venet et al. justified this issue by pointing to the operation of the proliferation signature, which considerably impacts a substantial portion of the genome. They suggested that most random gene sets contain some genes from the proliferation signature and, thus, are associated with the proliferation signature and, indirectly, survival. They defined the meta-PCNA signature to determine the proliferation rate and introduced a method to remove this signature’s impact on the expression data. They concluded that removing the effects of meta-PCNA genes on the expression of the genes in the NKI breast cancer dataset cohort was a perfect way to reduce the association between random genes and survival.

In 2018, Shimoni coined the term “random bias” to describe this concept in cancer^13^. Random bias is an unexpected situation in which more (less) than 5% of random gene sets are associated with some clinical attribute, such as survival time, in a statistically significant way. Shimoni examined The cancer genome atlas (TCGA) data for 34 different cancer types to see if there was a significant association between random gene sets and survival time. According to his analysis, random bias could be found in a wide variety of cancer types. Shimoni’s findings revealed that 17 out of the 34 datasets exhibited positive random bias, indicating that more than 5% of randomly selected gene sets in these cancers are significantly associated with survival time. Ten of these cancer types did not exhibit random bias, while seven of the datasets exhibited negative random bias, cancers with less than 5% significant survival-associated random (SSAR) gene sets. Shimoni utilized Venet’s approach to eliminating the confounder effect of the proliferation signature from TCGA expression data to reduce the effect of random bias in all types of cancer. His analysis concluded that Venet’s methods were ineffective in removing random bias in most cancer types and impractical in the TCGA breast cancer cohort. To solve this problem, Shimoni proposed that dividing samples into small subgroups using an unsupervised clustering method could decrease the proportion of SSAR gene sets in a wide range of cancer types. Shimoni’s results showed that out of the 106 clusters generated for all cancer types that had exhibited both positive and negative random bias, in only 65 of these clusters, the property of random bias was eliminated. Despite the fact that random bias was not eliminated in 41 of 106 cases, he contends that clustering can effectively eliminate random bias in several TCGA cancer types. Despite producing some promising and valuable insights, the existing research has produced contradictory results, is still limited in scope, and faces several critical theoretical and analytical challenges.

Previous studies have shown that significant survival-associated random gene sets can provide valuable insights into the biology of breast cancer and aid in identifying biologically cancer-related genes^14^. Notably, since random bias can be observed in many cancer types, it is possible that SSAR gene sets may also provide informative results for most cancer types. Building upon these findings, we assert that each cancer type has a fixed-point gene set that is biologically associated with cancer survival time which can be identified by SSAR gene sets. Additionally, these fixed-point gene sets are responsible for the observed random bias, and by removing their effects from expression data, it is possible to decrease the proportion of significant survival-associated random gene sets. To identify these fixed-point gene sets, we introduce an iterative novel approach for detecting gene sets that are not only statistically significant but also biologically relevant for cancer research and clinical practice. Specifically, we aim to identify fixed-point gene sets for each TCGA cancer type that exhibit positive random bias. By applying this approach, we aim to eliminate positive random bias and reduce the proportion of SSAR gene sets in a large number of cancer studies. Moreover, we demonstrate that the identified gene sets are highly biologically significant and can be considered as signatures for their associated cancer type. This suggests that the proposed approach can provide valuable insights into the underlying biology of cancer and improve the accuracy and reliability of survival analyses in various cancer types. Overall, our approach provides a systematic and rigorous method for detecting biologically relevant gene sets associated with cancer survival time and can have important implications for cancer research and clinical practice.

## Materials and methods

### Dataset

Loi et al.^15^ collected microarray expression data of 17,585 genes from 380 individuals with primary breast tumors. The Rdata file was downloaded from NCBI’s Gene Expression Omnibus (GEO) with accession number GSE6532 (https://www.ncbi.nlm.nih.gov/geo/query/acc.cgi?acc=gse6532).

#### Netherlands Cancer Institute (NKI) cohort

The NKI, also known as the van de Vijver et al. data set^5^, was provided in Venet’s paper^12^. This dataset contains microarray expression data of 13,108 genes for 295 breast cancer patients in stages I or II and their clinical data.

#### The Cancer Genome Atlas (TCGA)

The expression data of TCGA cancer types (17 cases) that exhibit positive random bias (based on Shimoni’s finding) were downloaded from the https://portal.gdc.cancer.gov site. We looked at level 3 data normalized using RNA-Seq by Expectation-Maximization (RSEM) method based on Shimoni’s approach; each dataset contains RNAseq expression datasets for cancer patients and their survival time and clinical data. We used standard TCGA study abbreviations for the cancer type names (as defined in https://gdc.cancer.gov/resources-tcga-users/tcga-code-tables/tcga-study-abbreviations).

### Method

In this section, we outline our novel approach for identifying the fixed-point gene set for each cancer type that exhibits the positive random bias property. Our method is based on an iterative algorithm to systematically and efficiently identify the gene set of interest. We explain the steps involved in our approach and provide a detailed description of how our method works to detect biologically relevant gene sets associated with cancer survival time.

#### Fixed-point gene set identifier method (FPGI)

**Figure 1:**
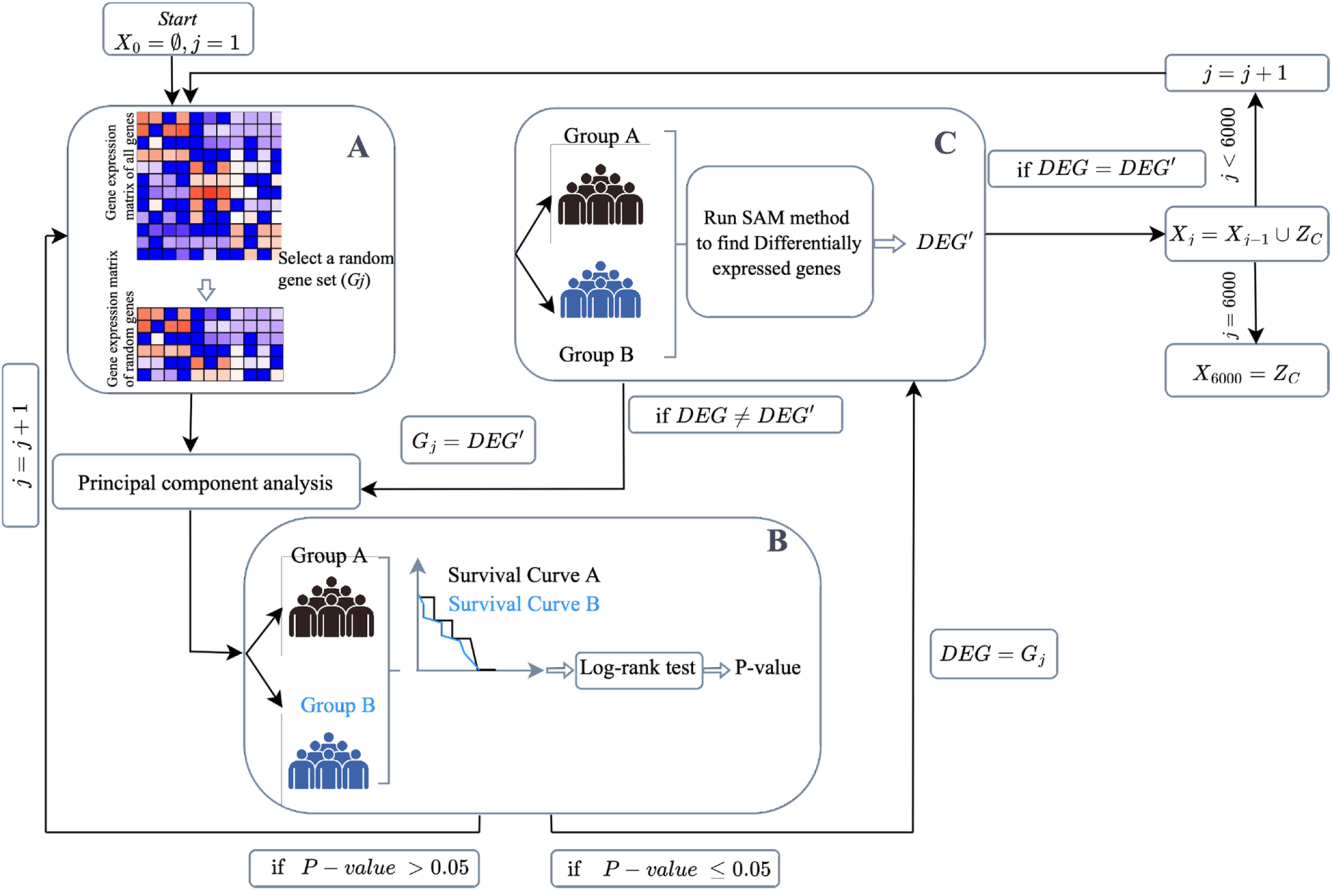
The figure depicts the FPGI method for identifying a fixed-point gene set in a given cancer type. The method starts with $j = 0$, and an empty set $X_{0}$ and proceeds iteratively for $j=1,\ldots ,6000$. At each iteration, a set of genes ($G_{j}$) is randomly selected from all genes expression data (GE all genes) and used to construct an gene expression data matrix (GE random genes) (part A). The samples are then divided into two groups *A* and *B* using principal component analysis (PCA), and their survival time is compared using log-rank test and *p*-value (part B). If the *p*-value is less than 0.05, set $DEG = G_{j}$ and the first 50 differentially expressed genes ($DEG'$) between *A* and *B* are identified using the Significance Analysis of Microarrays (SAM) method (part C). If *DEG* is the same as $DEG'$, it is added to the fixed-point set $X_{j}$, otherwise, the process is repeated with $DEG'$ as $G_{j}$ until convergence. In this method, *j* represents the iteration number. The final result, $X_{6000}$, is considered as the fixed-point set of the cancer type C.

1. Initialize $$X_0=\emptyset$$, $$j=1$$.
2. Randomly select a set of genes, $G_j$, with size *m* from all genes (Fig. 1A).
3. Use principal component analysis (PCA) on the gene expression data matrix of $G_j$ to divide samples into two equally sized groups (*A* and *B*) based on the median of the first principal component. Test the null hypothesis that there is no difference in survival time between these two groups using the log-rank test. If the *p*-value is less than 0.05, proceed to the next step. Otherwise, go back to step 2 and increase *j* by one and choose another random set ($G_{j+1}$) (Fig. 1B).
  - Set $DEG = G_j$. Use the Significant Analysis of Microarrays (SAM) method to detect differentially expressed genes between groups *A* and *B*, and consider the first *m* genes (most significant genes) as $DEG'$ (Fig. 1C).
  - Compare the sets $DEG'$ and *DEG*. If they were not the same, set $G_j = DEG'$ and go back to step 3.
  - If $DEG'$ and *DEG* were the same, set $X_j= X_{j-1}\cup DEG$ and go to step 4.
4. Increase *j* by one and go back to step 2.
5. Continue the whole process for $j= 1,\ldots ,6000$ and identify $Z_{C}= X_{6000}$ as the fixed-point gene set of cancer type C that exhibits positive random bias property.

#### Why FPGI method converges?

This iterative method tries to identify gene sets associated with positive random bias in cancer samples. This method combines Principal Component Analysis (PCA) to divide samples into two groups with Significant Analysis of Microarray (SAM) to identify differentially expressed genes between the two groups of samples resulting from PCA. This iterative process is repeated until a fixed set of genes is obtained. Utilizing the SAM method at each iteration ensures the convergence of our method, which helps to identify gene sets that are statistically significant and likely biologically relevant. In fact, the application of the SAM method during each iteration is the primary contributor to the method’s convergence. SAM is designed to identify gene sets with statistically significant differences in expression between two sample groups. Using PCA to refine the search for relevant gene sets is the second critical factor contributing to the convergence of the FPGI method. PCA is an efficient method for identifying sample subpopulations with potentially distinct gene expression profiles. By dividing the samples into two groups based on the identified gene set at each iteration, FPGI can identify subpopulations of samples with comparable gene expression profiles and narrow our search to the most relevant gene sets. In addition, the stopping criterion of this method, which requires that the gene set identified in each iteration be identical to the gene set identified in a previous iteration, ensures that the method does not continue to iterate forever.

In addition, the stopping criterion of this method, which requires that the gene set identified in each iteration be identical to the gene set identified in a previous iteration, ensures that the method does not continue to iterate forever. To investigate this, we tested our algorithm on different gene set sizes, including 5, 50, 100, and 200 genes. Our results show that for all scenarios, the algorithm converges quickly (with a maximum of 20 iterations).

The combination of SAM, and PCA, as well as identifying statistically significant gene sets at each iteration and refining the search based on the identified gene set, and the stopping criterion ensures that FPGI method converges on a fixed set of genes, that are biologically significant, making it a reliable and robust technique for identifying relevant gene sets in cancer samples.

#### Scoring function

Our scoring function, denoted as *w*, maps from the fixed-point gene set, $Z_{C}$ to the set of natural numbers, ${\mathbb {N}}$. Specifically, given a gene *g*, we define its score *w*(*g*) as the number of times *g* appears in the gene set $X_{j}$, where $X_{j}$ is computed in step 3 part c for $j=1,\ldots ,6000$. Therefore, *w*(*g*) provides a measure of the significance of each gene in $Z_{C}$ by quantifying its frequency of occurrence across iterations.

In the result section, we will discuss how the fixed-point set, $Z_C$ is associated with survival time and plays a key role in the phenomenon of positive random bias for the vast majority of cancer types exhibiting this property. Despite the fact that the results presented in this section pertain solely to BRCA, the same conclusions hold true for the other 16 types of cancer analyzed.

## Results

### Frequency of genes in fixed-point set

To ensure comprehensive coverage of the search space and the inclusion of all available genes in the dataset, we generated the union of 6,000 random gene sets, each containing 50 genes. This union resulted in a multi set of 300,000 genes. For the BRCA dataset, the union set contained all of the 18,275 genes of the dataset at least once, and some genes were repeated up to 34 times. Thus, our iterative method, which starts from a random gene set and repeats 6000 times, covers all genes in the dataset, giving each gene a chance to be chosen in a random gene set.

On the other hand, the fixed-point set of BRCA ($Z_{\textrm{BRCA}}$) consists of only 295 genes, with a maximum and minimum frequency of 397 and 52 respectively. Compared to the original dataset, this is a very small set of genes. These results demonstrate that our method attempts to cover the entire search space and starts from all available genes in the dataset for each cancer type. Eventually, it settles on a small subset of genes that is a subset of the corresponding cancer type genes.

**Figure 2:**
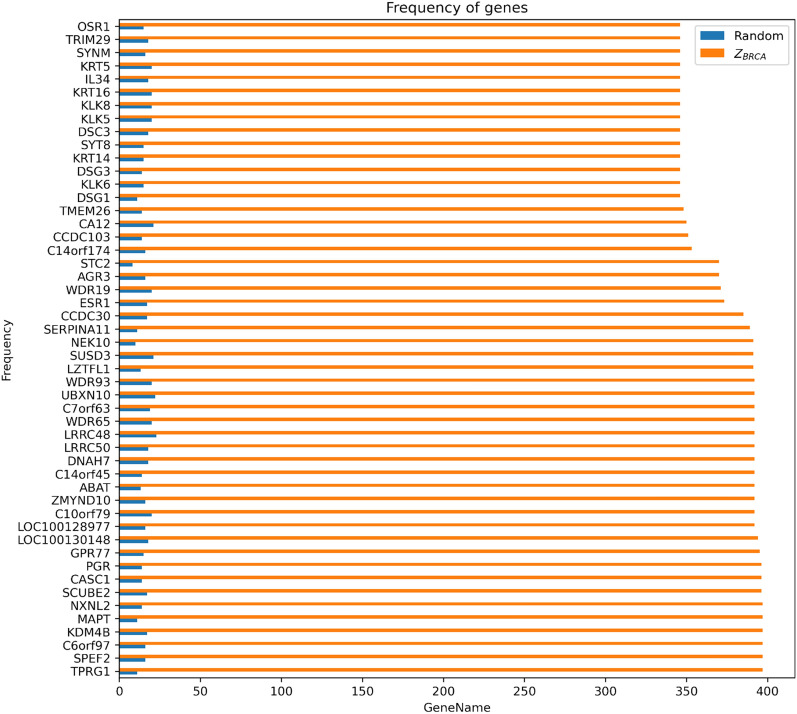
Frequency of genes in all random gene sets versus scores of the fixed-point gene set. The orange bars on the y-axis represent the 50 top scoring genes in the breast. In random gene sets, the scoring value of these genes is represented by blue bars.

The scoring values for the 50 top genes in $Z_{\textrm{BRCA}}$ are shown in Fig. 2. We choose this number of genes for the figure because we mainly report our results using a random gene set size of 50 throughout most of the paper, even though we analyzed our method using random gene sets of size 50, 100, and 200. It is noteworthy that the scores of the top 50 genes in $Z_{BRCA}$ ranged from 346 to 397. The frequencies of these genes in the random gene sets and in $Z_{\textrm{BRCA}}$ are plotted in blue and orange bars, respectively. Similar plots for other cancer types are available in Supplementary Information File 1, and they follow the same pattern.

Overall, we observe that the $Z_{\textrm{C}}$ set is significantly smaller than the original dataset. This suggests that the genes in the $Z_{\textrm{C}}$ set may be important for cancer progression. To investigate this claim, we analyze the biological relevance of $Z_{\textrm{C}}$ to cancer *C* in the following sections.

### Protein-protein interaction (PPI) network and pathway enrichment analysis

**Figure 3:**
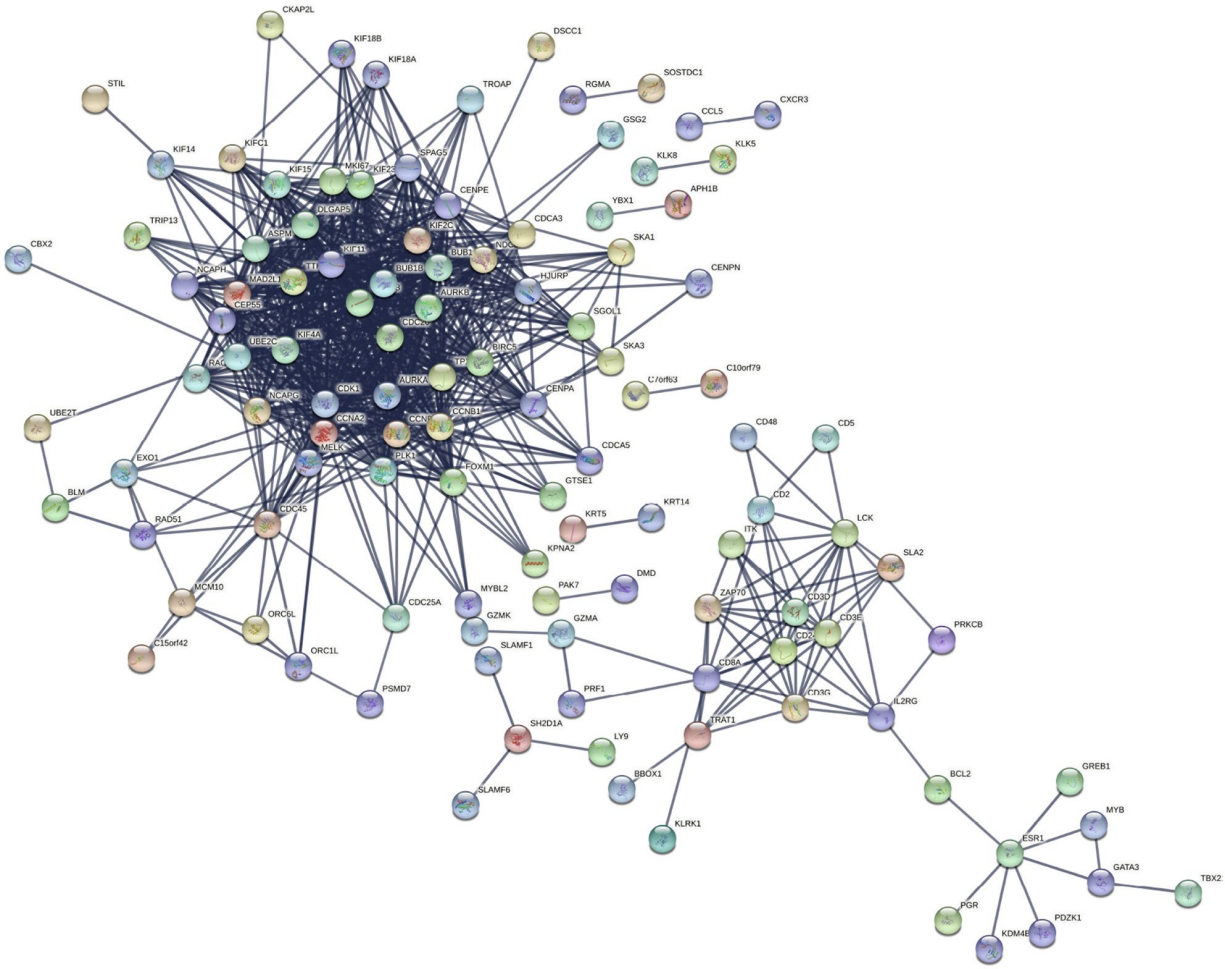
STRING protein-protein interaction analysis of the fixed-point gene set of the BRCA dataset. The network contained 113 nodes and 810 edges (vs. 84 expected edges); enrichment *p*-value less than 1.0*e* $-$16. Figure were constructed using the STRING database (version 11.5; https://string-db.org/).

In the first step of our analysis, in order to determine functional interactions between proteins coding genes of the resulted $Z_{\textrm{C}}$, we used the Search Tool for Retrieval of Interacting Genes (STRING) database https://string-db.org^16^. The PPI network was constructed using active interaction sources such as text mining, experiments, databases, neighborhood, gene fusion, co-occurrence, and co-expression, and a species was restricted to “Homo sapiens”. The nodes in the network represented the proteins, while the edges reflected the interaction. In STRING, each protein-protein interaction is annotated with one or more ‘scores’, these scores are indicators of confidence. Each score is assigned a confidence level between 0 and 1, with 1 representing the highest level of confidence. To obtain more reliable findings, we used a score of 0.9 for the confidence of interactions. The $Z_{\textrm{BRCA}}$ gene-based network consisted of 113 non-isolated nodes and 810 edges. The constructed network has a PPI enrichment *p*-value less than 1.0*e*−16, indicating that interactions between genes were not random. This suggested that the $Z_{\textrm{BRCA}}$ genes interacted more frequently than would be predicted for a random collection of proteins with the same size and degree distribution (in this case expected number of edges is 84). This enrichment indicated that the proteins as a group are biologically related. As it is illustrated in Fig. 3, the network, which was reduced to none isolated nodes contained some distinct, dense modules. Similar results were observed for other cancer types, which are available in Supplementary Information File 2. These results indicated that the genes in the $Z_{\textrm{C}}$ sets were highly associated with one another.

Accordingly, in the second step, Kyoto Encyclopedia of Genes and Genomes (KEGG) pathway enrichment analyses were conducted using The Database for Annotation, Visualization and Integrated Discovery (DAVID) (https://david.ncifcrf.gov) to find corresponding significant pathways of $Z_{\textrm{C}}$ for each of the 17 cancer types^17,18^. Supplementary Information Fig. 1 shows the significant pathways common in at least two types of cancer. In this figure, the names of the pathways are listed in the first column, and the pathways associated with each cancer type are depicted in the second column using different colors. As presented in Supplementary Information Table 1, most of the significantly enriched pathways of $Z_{\textrm{C}}$ were highly associated with cancer *C*.

### Association of fixed-point set with disease

The Genetic Association Database (GAD) tool on the David Functional Annotation server (https://david.ncifcrf.gov) was utilized to investigate the association between $Z_{\textrm{C}}$ genes and disease. GAD is a database of published genetic association studies that enable the investigation of complex common human genetic diseases^17,18^. Table 1 demonstrates the enriched disease and the disease class for each of the 17 cancer types. The table suggested that the genes in $Z_{\textrm{C}}$ were associated with cancer C. For instance, in the case of BRCA, the top-level disease class and disease assigned by GAD were cancer and breast cancer with *p*-values of 2.8*e* $-$4 and 6.5*e* $-$8, respectively. As noted by Ansar et al.^14^, these findings indicated how our method can detect meaningful information in SSAR gene sets.

**Table 1:** Enriched disease and disease class achieved from fixed-point sets by Genetic Association Disease (GAD).

Dataset	GAD disease class	Class *p*-value	GAD disease	*p*-value
ACC	Cancer	2.2*e* $-$3	Plasma HDL cholesterol (HDL-C) levels	9.9*e* $-$6
BLCA	Cancer	9.2*e* $-$6	Urinary bladder neoplasms	1.8*e* $-$2
BRCA	Cancer	2.8*e* $-$4	Breast cancer	6.5*e* $-$8
GBMLGG	Cancer	2.8*e* $-$4	Schizophrenia	2.0*e* $-$6
HNSC	cardiovascular	1.6*e* $-$5	Cardiomyopathy, Dilated|DCM—Dilated cardiomyopathy	3.7*e* $-$6
KIPAN	Cancer	7.5*e* $-$4	Type 2 Diabetes| edema | rosiglitazone	7.5*e* $-$5
KIRC	Cancer	2.5*e* $-$6	Chronic renal failure|Kidney failure, Chronic	1.9*e* $-$3
KIRP	Cancer	3.1*e* $-$5	Type 2 Diabetes| edema | rosiglitazone	9.6*e* $-$6
LGG	Pharmacogenomic	2.3*e* $-$5	Several psychiatric disorders	2.0*e* $-$6
LIHC	Cancer	9.0*e* $-$5	Liver cancer	6.1*e* $-$2
LUAD	Cancer	2.4*e* $-$17	Lung cancer	2.0*e* $-$6
LUSC	Cancer	9.3*e* $-$4	Lung Diseases|Resp distress syndrome neonatal	2.1*e* $-$6
MESO	Cancer	1.1*e* $-$3	Lung cancer	2.5*e* $-$2
PAAD	Cancer	1.5*e* $-$2	Type 2 Diabetes| edema | rosiglitazone	8.2*e* $-$4
THYM	Immune	1.6*e* $-$2	Pulmonary disease, Mycobacterium malmoense	1.5*e* $-$4
UCEC	Cancer	2.0*e* $-$4	Dermatitis, Atopic	1.3*e* $-$6
UVM	Cancer	1.3*e* $-$2	Uveitis	3.8*e* $-$2

### Association of top scoring genes of fixed-point set with cancer *C*

Through pathway enrichment, PPI network analysis, and disease class association, the biological significance of $Z_{\textrm{C}}$ with respect to its corresponding cancer type was investigated in the previous sections, and it was determined that the obtained fixed-point gene sets were significantly associated with cancer progression and metastasis. Although, the most significant advantage of our method was that the high-scoring genes of $Z_{\textrm{C}}$ in many studies had been shown as cancer driver genes. In the ACC, *CD*68 gene, the highest-scoring gene in $Z_{\textrm{ACC}}$, has been identified as a prognostic biomarker for adrenocortical carcinoma^19^. As another example, the *TBX*2 and *TBX*3 genes, with scores of 1276 and 1226 in the fixed-point gene set of BLCA, were excellent markers for predicting progression to muscle-invasive bladder cancer in patients with primary pTaG1/2 bladder cancer^20^. In^21^, it has been proposed that the *C*6*orf*97 gene, the highest scoring gene of $Z_{\textrm{BRCA}}$, might play important roles not only in carcinogenesis but also in the progression of breast cancer patients toward a more aggressive phenotype. In 2019, Yeng et al.^22^ had indicated that *ACTA*1, a gene from $Z_{\textrm{HNSC}}$ with a score of 245, was a biomarker of head and neck squamous cell carcinoma. As another instance, silencing of *ANK*2 with a score of 358 in $Z_{\textrm{PAAD}}$ decreased the proliferation of the pancreatic tumor cells and reduced their tumorigenicity in vitro and in vivo^23^. The highest-scoring genes across all $Z_{\textrm{C}}$ sets are depicted in Supplementary information Fig. 2 and, Published papers that have investigated their association with cancer are available in Supplementary information Table 2.

### Random bias correction

The random bias phenomenon, as described by Venet et al. and Shimoni, suggested that many of the signatures identified in numerous analyses of cancer types might not be causal of cancer progression, despite their significant association with survival time. Consequently, random bias is a confounding property that must not be ignored^12,13^. As proposed by Venet et al. random bias was caused by the activity of proliferation genes (meta-PCNA gene signature) in data that had a substantial impact on the expression data, and the activity of this signature significantly influences each random set selected from the data. They hypothesized that by removing the effect of meta-PCNA genes from expression, the random bias in the NKI breast cancer dataset could be eliminated^12^. However, Shimoni demonstrated that removing the impact of meta-PCNA genes could not effectively reduce the proportion of SSAR gene sets in TCGA cancer types. Venet’s strategy might depend on the platform or data^13^. In this paper, we claimed that for each cancer type, we required a specific set of genes that removing its impact on the expression data could reduce the proportion of SSAR gene sets. To investigate this claim, we used the fixed-point gene set ($Z_{\textrm{C}}$) of each cancer type and demonstrated that removing the influence of these genes from expression data could dramatically reduce the proportion of SSAR gene sets in the vast majority of cancer types. To accomplish this, we selected 10% of the highest scoring genes of $Z_{\textrm{C}}$ and then removed their impact from the expression data similar to Venet’s approach^12^. The result of this analysis is available in Table 2, where rows denote the cancer type and the proportion of significant *p*-value in percentage (SSAR%), the proportion of significant random gene set after removing the effect of meta-PCNA in percentage (PCNA-SSAR%), and the proportion of significant SSAR gene set after removing the effect of corresponding $Z_{\textrm{C}}$ ($Z_{\textrm{C}}$-SSAR) are collected in first, second and third columns, respectively. As shown in Table 2, the proportion of significant (positive random bias) in 14 out of 17 cancer types was significantly more reduced by using the selected genes from $Z_{\textrm{C}}$ rather than meta-PCNA.

**Table 2:** The proportion of significant survival associated random gene sets after removing the fixed-point set and meta-PCNA signature.

Dataset	SSAR%	PCNA-SSAR%	$Z_{\textrm{C}}$-SSAR%
ACC	71	40	5
BLCA	50	44	7
BRCA	21	18	5
GBMLGG	99	85	25
HNSC	26	27	20
KIPAN	64	26	27
KIRC	82	68	26
KIRP	57	17	5
LGG	80	64	19
LIHC	32	5	7
LUAD	49	18	11
LUSC	14	10	6
MESO	53	20	15
PAAD	45	7	13
THYM	16	18	7
UCEC	58	41	27
UVM	51	45	7

“SSAR%” is the proportion of significant survival-associated random gene sets, “PCNA-SSAR %” is the proportion of significant survival-associated random gene sets after removing the effect of meta-PCNA signature from their expression data, and “$Z_{\textrm{C}}$-SSAR%” is the proportion of significant survival associated random gene set after removing the effect of $Z_{\textrm{C}}$ gene set from expression data.

**Figure 4:**
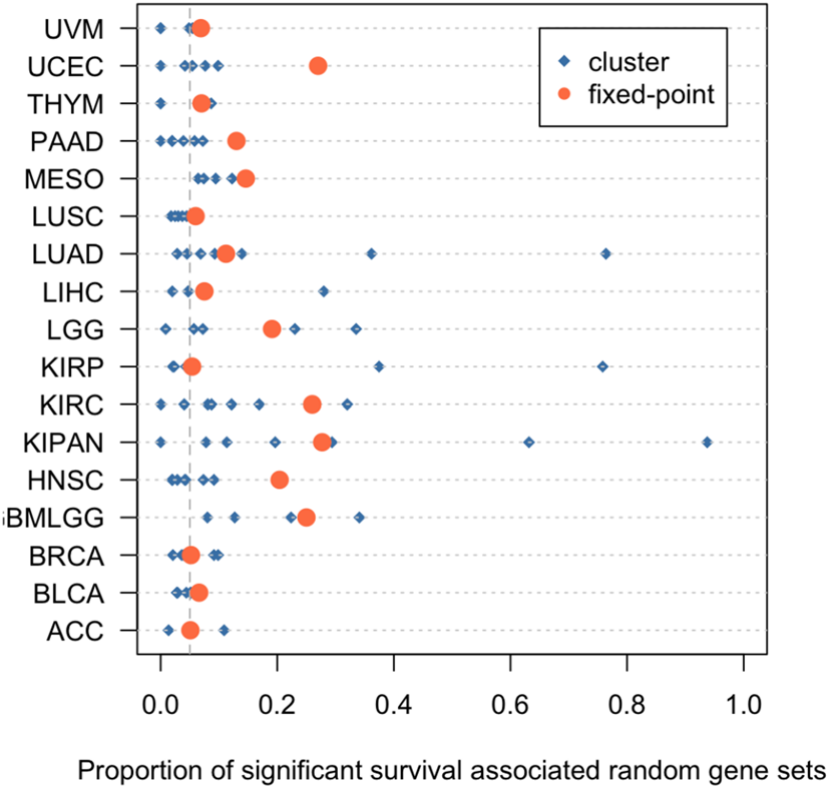
Each horizontal line represents a TCGA cancer type with positive random bias property. Each dot along the x-axis represents the proportion of significant survival-associated random gene set in each cluster. The short vertical gray line illustrates the proportion of significant survival-associated random gene set after removing the effect of $Z_{\textrm{C}}$ genes from expression data.

From another point of view, Shimoni used the PhenoClust, an unsupervised clustering method, to reduce the effect of random bias in TCGA cancer types^13,24^. In Shimoni’s approach, the samples of each cancer type have been divided into sub-clusters, the association between each random gene set and the survival time of each cluster’s samples have been determined, and the proportion of SSAR gene sets was calculated for each cluster. Samples of all 17 cancer types with positive random bias property have been divided into 92 clusters^13^. In Fig. 4 proportion of SSAR gene sets is represented with colored dots and the small horizontal grey lines show the proportion of SSAR gene sets after removing the effect of corresponding $Z_{\textrm{C}}$. Our evaluation of the performance of the proposed method was based on the assumption that a method is considered perfect for removing the random bias property if the results of the method cause only 0.05 of the random sets to remain significant. We compared the performance of our proposed method, FPGI, with that of Shimoni’s clustering method using the equation presented in the Supplementary Information File 3, where $SSAR_{\textrm{CL}}(i)$ denotes the proportion of significant random gene sets of cluster *i* of cancer *C*, and $N_{\textrm{C}}$ represents the number of generated clusters for the corresponding cancer type. We evaluated the distance between the proportion of SSAR gene sets after excluding the effect of 10% of the highest scoring genes of $Z_{\textrm{C}}$ from the expression data, denoted by $DZ_{\textrm{C}}$, to demonstrate the effectiveness of our method. The results of this analysis are presented in Supplementary Information File 3 table. This table indicates the cancer types with positive random bias property in the first column, $AD_{\textrm{C}}$ for each cancer type in the second column, and $DZ_{\textrm{C}}$ in the third column. Our results demonstrate that, in 9 out of 17 cancer types, $DZ_{\textrm{C}}$ is less than $AD_{\textrm{C}}$, which indicates that our proposed method outperforms Shimoni’s clustering method. In 5 out of the 8 remaining cancer types the results of these two methods are comparable^12,13^. While it is true that our method shows better results compared to a specific cluster and not all clusters, we believe that this comparison still provides valuable insight into the performance of our proposed method. Moreover, our proposed method provides an alternative explanation to the same problem and also provides a significant set of genes to continue exploration.

### Reproducibility

In cancer research, identifying a reliable gene set independent of datasets and can accurately predict a patient’s survival outcome has become a major obstacle. Numerous articles on discovering survival-relative genes in various cancer types have been published over the recent decades, each proposing a gene set, with the authors asserting that the purpose gene set was significantly associated with cancer progression and metastasis. However, there was little overlap between the gene sets resulting from studies with different cohorts but similar analytic methods. In this paper, we introduced a set of significant survival-relative genes and indicated that we could reduce the proportion of SSAR gene sets by employing them. We demonstrated that these finding sets were nearly robust across different cancer cohorts. For this reason, we evaluated our results using various breast cancer cohorts and data sets. Regarding this, we considered three distinct breast cancer cohorts (NKI, TCGA, and LOI)^5,15^. For the NKI, TCGA, and LOI cohorts, the resulting $Z_{\textrm{C}}$ sets contained 364, 295, and 426 genes, respectively. Each pair of these three gene sets has more than 30% of their genes in common, and 37 genes are in the intersection of all $Z_{\textrm{C}}$ sets that contained critical genes for breast cancer like *AURKA* and *AURKB*^25,26^. Also, the PPI network based on these 37 genes was very dense, and the corresponding pathways of these genes were significantly associated with breast cancer (see Supplementary Information Fig. 3). It could be concluded that our proposed method for identifying survival-associated genes demonstrated significant overlap across different cohorts.

## Discussion

It has previously been shown that gene expression data from random gene sets have a significant relation with cancer survival time, which has been discovered in a variety of cancers. Venet et al.^12^ first discovered this phenomenon in a microarray-measured expression dataset in breast cancer. As it turns out, this pattern can be seen in nearly all of the TCGA data matrix’s RNAseq-derived gene expression data. Venet et al.^12^ hypothesized that the phenomenon of random bias observed in gene expression data is caused by the activity of the proliferation signature, which affects a substantial portion of the human genome, and by removing the effect of this signature from expression data the random bias property will be removed. However, As reported by Shimoni^13^, the proliferation signature alone is insufficient to eliminate this bias in most cancers. While we agree with the general assumption of Venet et al. we believe that the specific set of genes that contribute to positive random bias varies widely between cancer types.

To address this issue, we propose the existence of a fixed-point gene set for each type of cancer, which exerts a strong influence on a large number of genes in the genome and is strongly associated with survival time. This fixed-point gene set can induce survival prediction ability in randomly selected gene sets derived from expression data. To identify these gene sets, we developed an innovative and iterative method .

The iterative nature of our method is a key strength that enables us to identify gene sets that are not the result of chance or noise, but instead represent significant differences in gene expression between groups. This is achieved by repeatedly dividing samples into two groups based on the differential expression of a gene set, and using this information to identify a new, refined gene set. The iterative process enables us to focus on the most biologically relevant genes for a given cancer type, and exclude genes that may be false positives or irrelevant to the disease.

By applying this method to multiple cancer types, we can build a more comprehensive understanding of the underlying molecular mechanisms driving cancer development and progression. Furthermore, because our method is based on statistical significance, we can be confident that the gene sets we identify reflect genuine differences in gene expression between cancer types. This in turn gives us greater confidence in the biological relevance of the genes we identify, and increases the potential for these genes to be used as diagnostic or therapeutic targets in the future.

In our study, we have analyzed a wide range of cancer types, and in order to validate the biological relevance of the identified gene set, we conducted protein-protein interaction (PPI) network and pathway analyses. The PPI network analysis helped us identify key biological pathways and processes involved in cancer development and progression and how the fixed-point set was related to these pathways. We also compared our gene set with previously published cancer signatures and confirmed that our identified gene set was highly correlated with the known cancer pathways.

In addition, we evaluated the association of our fixed-point set with cancer disease class and related cancer types using the Genetic Association Database (GAD). The results showed that our gene set was highly associated with cancer disease class and related cancer types, providing further evidence of the biological relevance of our identified genes.

To ensure that our findings were not dataset-specific, we reanalyzed our method with other independent datasets. The results showed that our identified fixed-point set of genes was consistently present across different datasets, further validating our approach and increasing the confidence in our results.

Overall, our study has identified a set of highly cancer-related genes using an iterative approach that eliminates false positives and ensures the biological relevance of the identified genes. The validation of our gene set through various approaches and its consistency across multiple cancer types and datasets further supports its potential as a diagnostic or therapeutic target for cancer treatment.

FPGI method is inspired by Brouwer’s fixed-point theorem, to identify this fixed-point gene set. Brouwer’s fixed-point theorem states that if you have a continuous function *f* that maps a compact, convex set *X* onto itself, and function *f* is a contraction, that it reduces distances between points in *X* by a constant between 0 and 1, then there is always a point *Z* in *X* such that $f(z) = Z$. In other words, *X* contains a fixed point that does not move as a result of the function *f*. In this work, we drew inspiration from this theorem by considering the set of all subsets of size m from all genes to be the compact, convex set *X*, the symmetric difference of the sets to be the metric on *X*, and the composition of the SAM and PCA methods to be the continuous function on *X*.

To create this method, we first use a technique for detecting a significant relationship between gene expression data and cancer sample survival time in order to divide the group into two equal-sized subgroups with different survival dynamics. Second, we use a method to find genes whose expression significantly differs between these two subgroups.

We used PCA to estimate the association between a randomly chosen set of genes and survival time. Specifically, we calculated the median of the first principal component, and then based on its values the patients were divided into two equaled-sized groups (A and B). The use of PCA as a method to estimate the association between a gene set and survival time has been previously validated in the literature, and we chose this approach because based on Venet’s results PCA method has been shown to reveal stronger outcome associations than other methods such as, kmeans and hierarchical clustering^27–33^. Moreover, the use of PCA in this method allows for a more efficient and effective identification of genes that are relevant to patient survival, as it reduces the number of variables needed to analyze the data and highlights the most important variables. In summary, PCA is a widely used statistical technique that we used to estimate the association between a randomly chosen set of genes and survival time. The use of the first principal component as a prognostic score has been validated in the literature and has been shown to reveal stronger outcome associations than other methods. In addition, there are many well-known methods to detect differentially expressed genes between two different groups. Recent studies showed that our method of choice (SAM methods) is a stronger approach^2^.

As mentioned in the result section, in 8 out of 17 cancer types by removing the effect of fixed-point, positive random bias was removed and the number of random significant gene sets was reduced to a significant level of 5% and in other 9 datasets the proportion of SSAR gene set was dramatically reduced. For instance, in case of GBMLGG it was reduced from 99 to 26%. In general, as explained in previous sections, various studies proposed a set of genes that are significantly associated with cancer progression and metastasis; however, the fact that many random gene sets may exist with similar association undermine their identity. We observed that our suggested sets are truly causal, in the sense that altering their expression or activity will influence survival where by removing the effect of these genes’ expression from our dataset, the association of such random set with survival, is eliminated. Since the choice of association method and also methods for detecting significant genes is of course crucial and has a substantial impact on our results, we can examine alternative methods to enhance our findings in future research.

To assess the robustness of our method, we conducted additional experiments by running our algorithm on random gene sets of size 100 and 200 for BRCA cancer. We observed that our method consistently identified nearly the same set of genes as the fixed-point set, regardless of the size of the set. Specifically, we found a high degree of overlap between the fixed-point sets obtained for sizes 50, 100, and 200, demonstrating the robustness and consistency of our method to the choice of random gene set size. These results indicate that our method is robust and reliable for identifying the fixed-point gene set in different random gene sets of varying sizes. The details of this analysis are provided in the Supplementary Information File 4. The file contains a figure and three tables that compare the selection of random gene sets of size 50, 100, and 200 genes for the fixed-point analysis. Tables of the Supplementary Information File 4 report the top ten highest scoring genes of $Z_{BRCA}$ identified by random gene sets of sizes 50, 100 , and 200, respectively. In addition, figure of the Supplementary Information File 4 illustrates the proportion of significant survival associated random gene sets before and after removing $Z_C$.

## Conclusion

Our study introduces a novel method that utilizes significant survival-associated random gene sets to identify a fixed-point gene set for cancers with positive random bias. The aim of this method is to identify a small set of genes specific to each cancer type that significantly affects survival time, referred to as the fixed-point gene set. This gene set remains stable across different random gene sets and serves as a core biological process underlying cancer progression for each specific cancer type. We expect our algorithm to converge to a similar fixed-point gene set that consistently affects survival time in different random samples from the same cancer type. Our approach combines Principal Component Analysis (PCA) and Significance Analysis of Microarrays (SAM) methods to reduce noise and approach the fixed-point gene set in each iteration. The empirical results on multiple cancer types demonstrate that our method effectively eliminates the random bias and improves the accuracy of survival prediction in gene expression data.

Our study also highlights the biological significance of the $Z_{\textrm{C}}$ genes and their association with cancer-related pathways. By using multiple studies, we show that the highest-scoring $Z_{\textrm{C}}$ genes are strongly associated with the progression and metastasis of their respective cancer types. Removing the effect of 10% of the highest-scoring genes on $Z_{\textrm{C}}$ from the expression data drastically reduces the proportion of random significant survival-associated gene sets and, in some cases, eliminates the positive random bias phenomenon.

## Supplementary Information

Supplementary Figure 1.

Supplementary Figure 2.

Supplementary Figure 3.

Supplementary Information 1.

Supplementary Information 2.

Supplementary Information 3.

Supplementary Information 4.

Supplementary Table 1.

Supplementary Table 2.

## Data Availability

The datasets and codes can be found in the GitHub repository https://github.com/maryammagy/FPGI.
