# Supplementary figures and images for: Removing the association of random gene sets and survival time in cancers with positive random bias using fixed-point gene set
Source: Sci Rep. 2023 May 29;13:8663. doi: 10.1038/s41598-023-35588-5 (PMC10226989; doi:10.1038/s41598-023-35588-5)

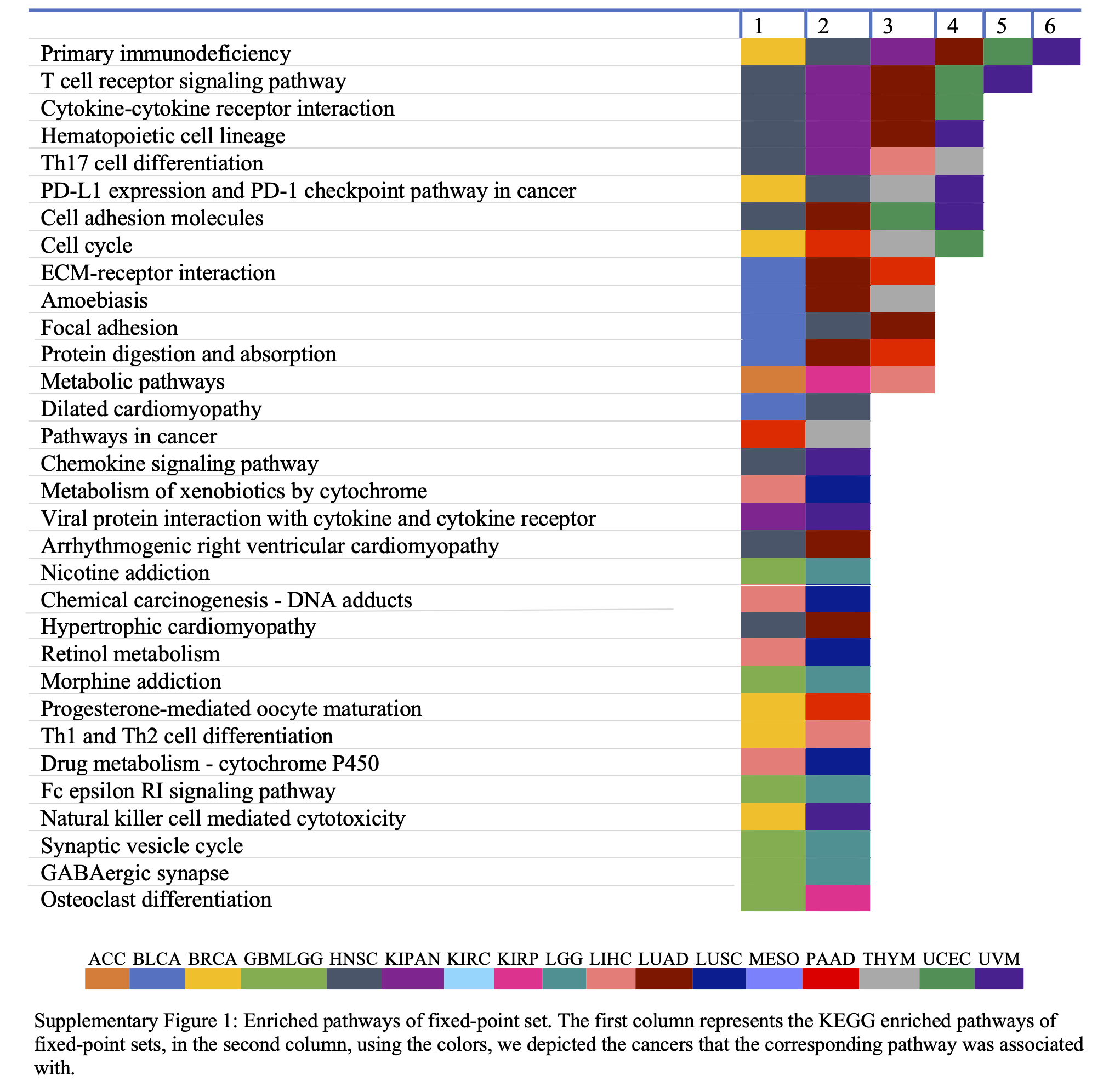

Supplement: Supplementary file 1 — Supplementary Figure 1. [file 41598_2023_35588_MOESM1_ESM.tiff]

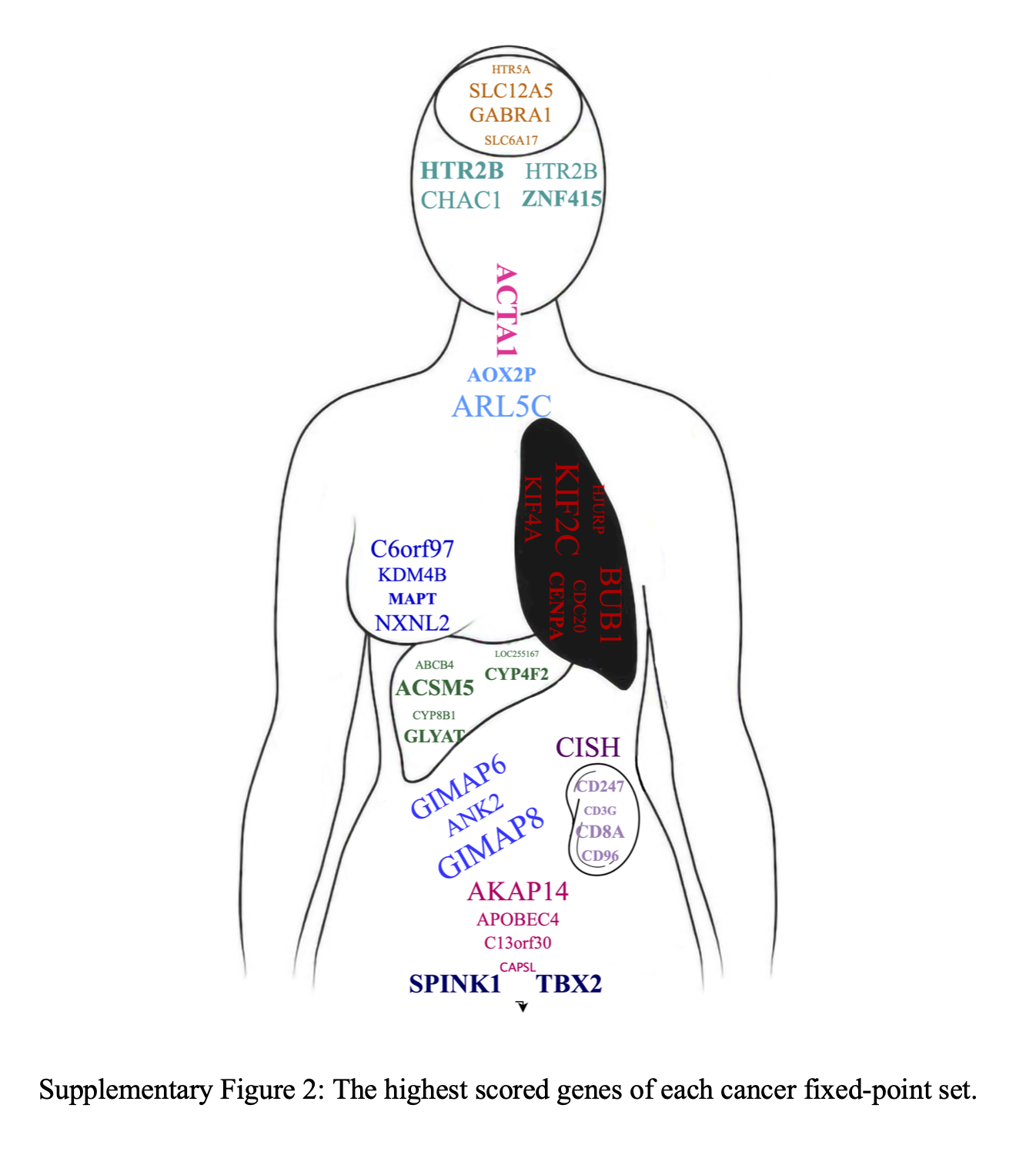

Supplement: Supplementary file 2 — Supplementary Figure 2. [file 41598_2023_35588_MOESM2_ESM.tiff]

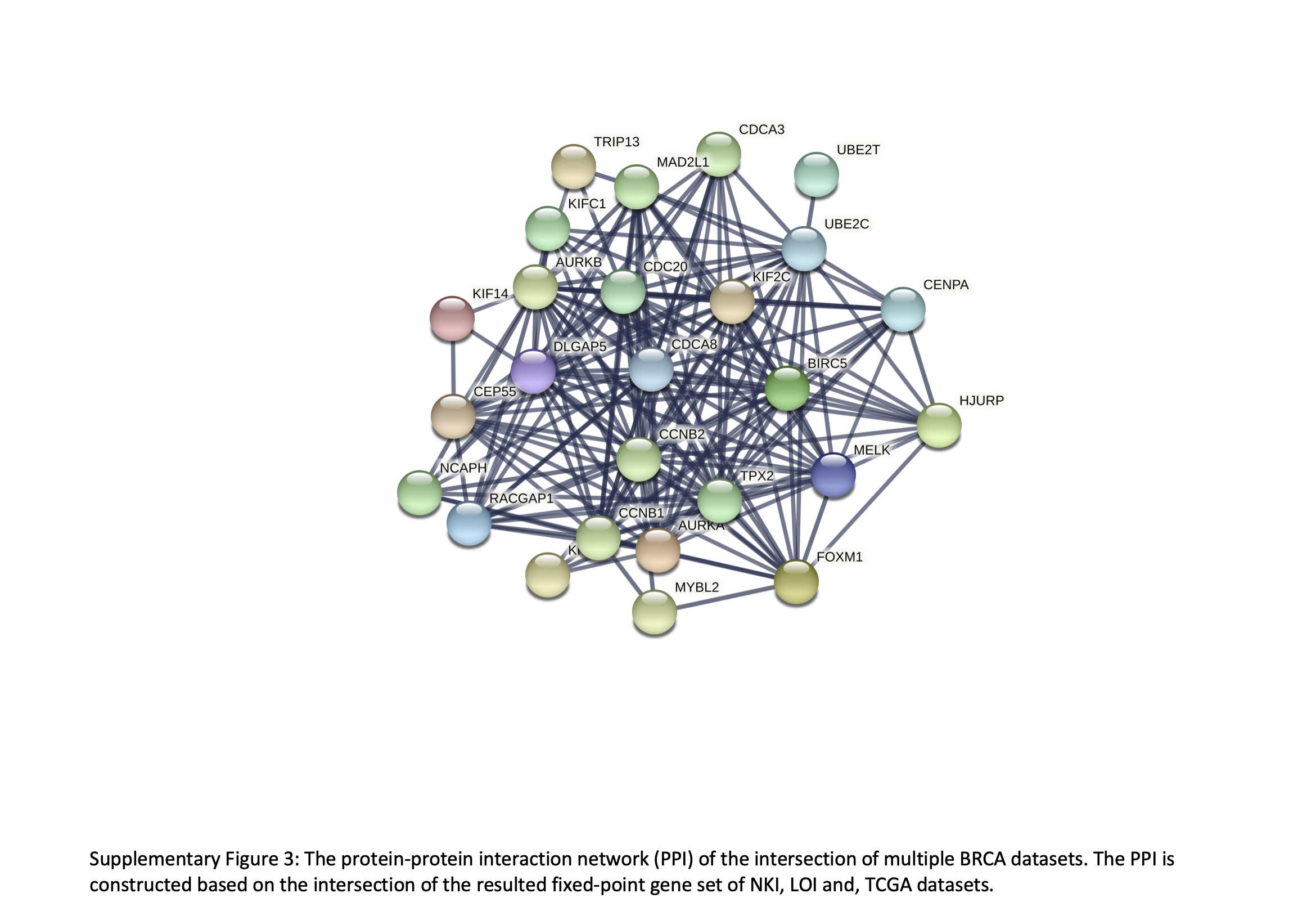

Supplement: Supplementary file 3 — Supplementary Figure 3. [file 41598_2023_35588_MOESM3_ESM.tiff]
