# Supplementary material for: Removing the association of random gene sets and survival time in cancers with positive random bias using fixed-point gene set
Source: Sci Rep. 2023 May 29;13:8663. doi: 10.1038/s41598-023-35588-5 (PMC10226989; doi:10.1038/s41598-023-35588-5)
Supplement: Supplementary file 4 — Supplementary Information 1. [file 41598_2023_35588_MOESM4_ESM.pdf]

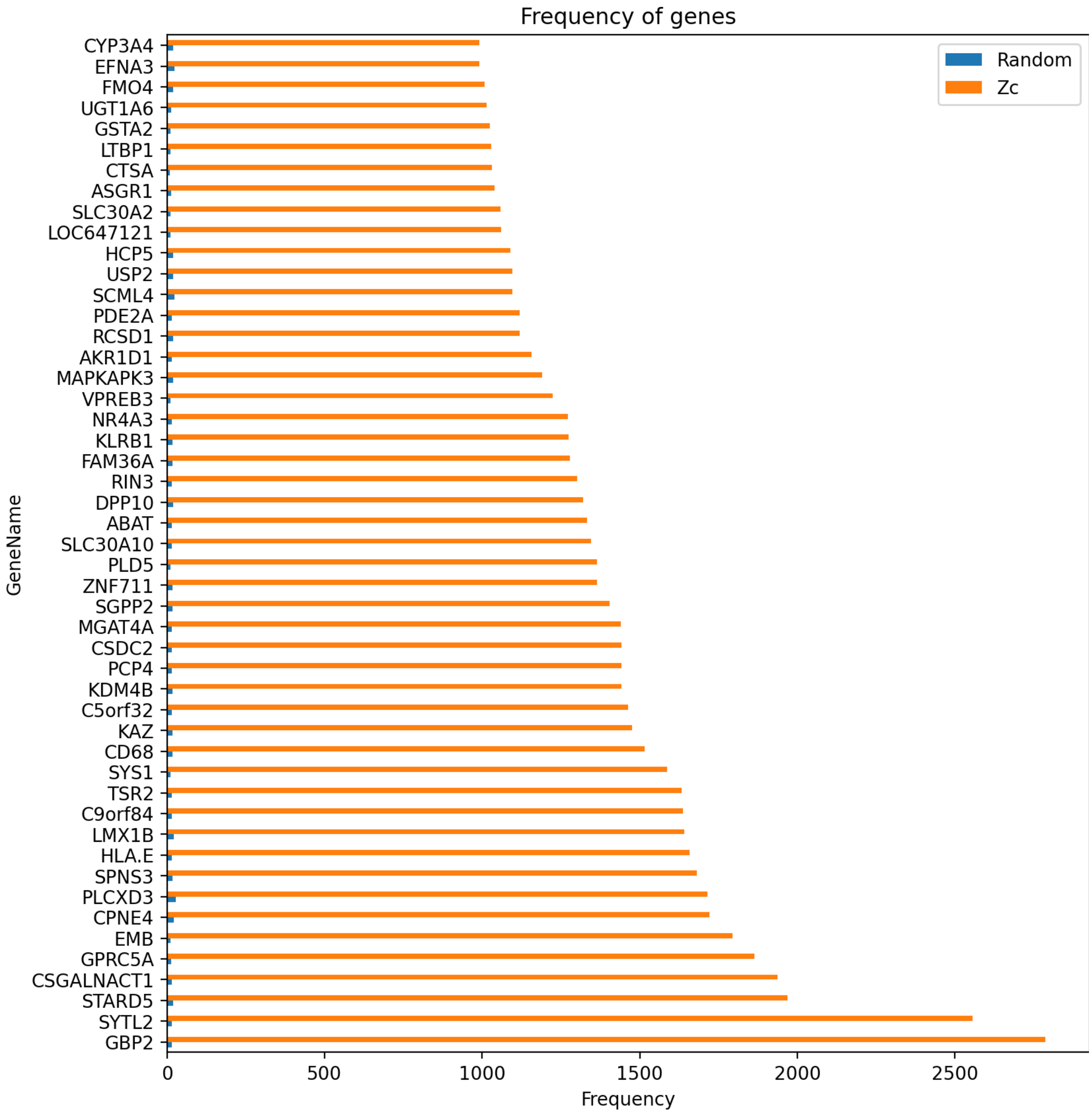

Frequency of genes in all random gene sets vs. scores of the fixed-point gene set of ACC

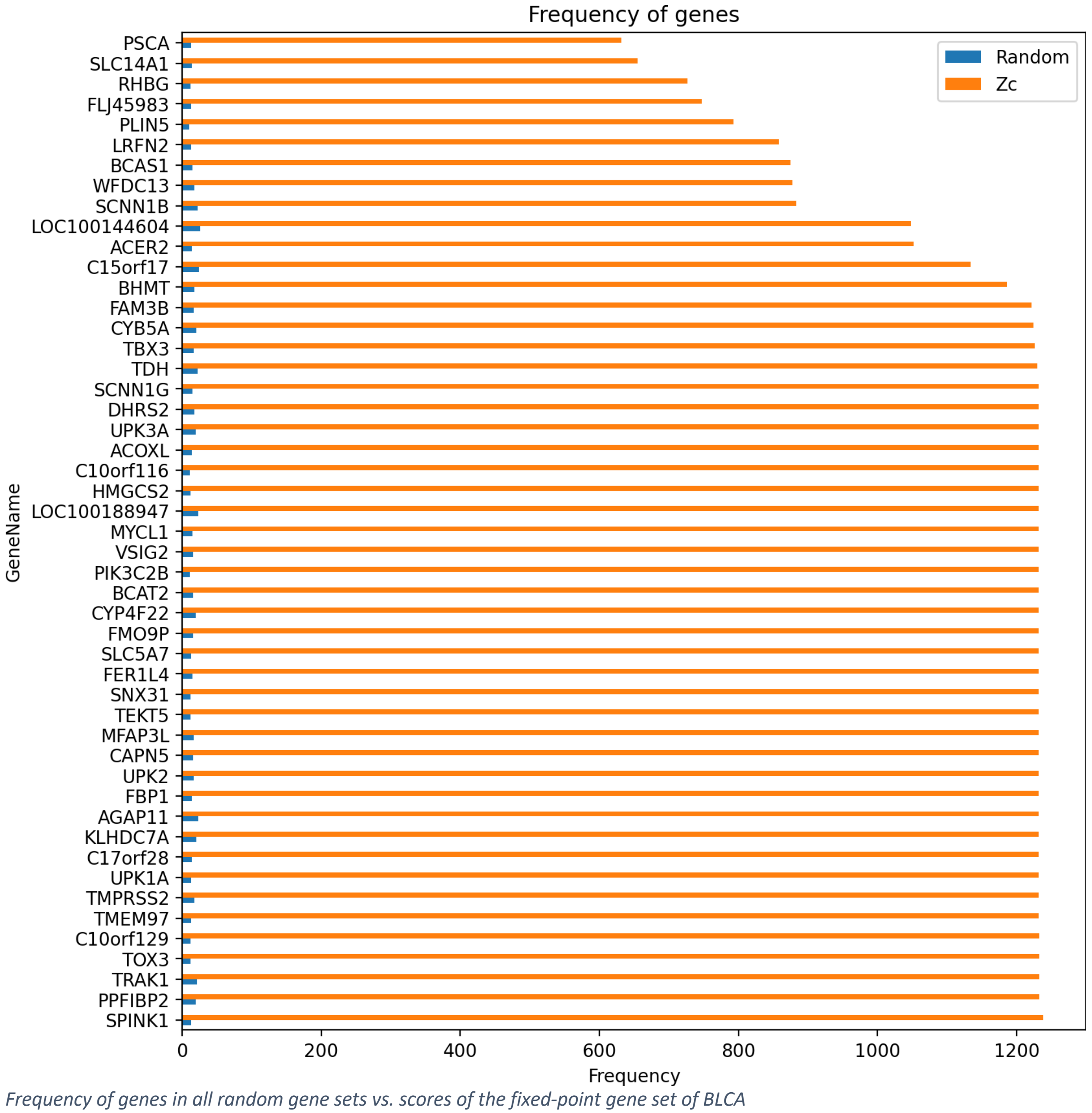

Frequency of genes in all random gene sets vs. scores of the fixed-point gene set of BLCA

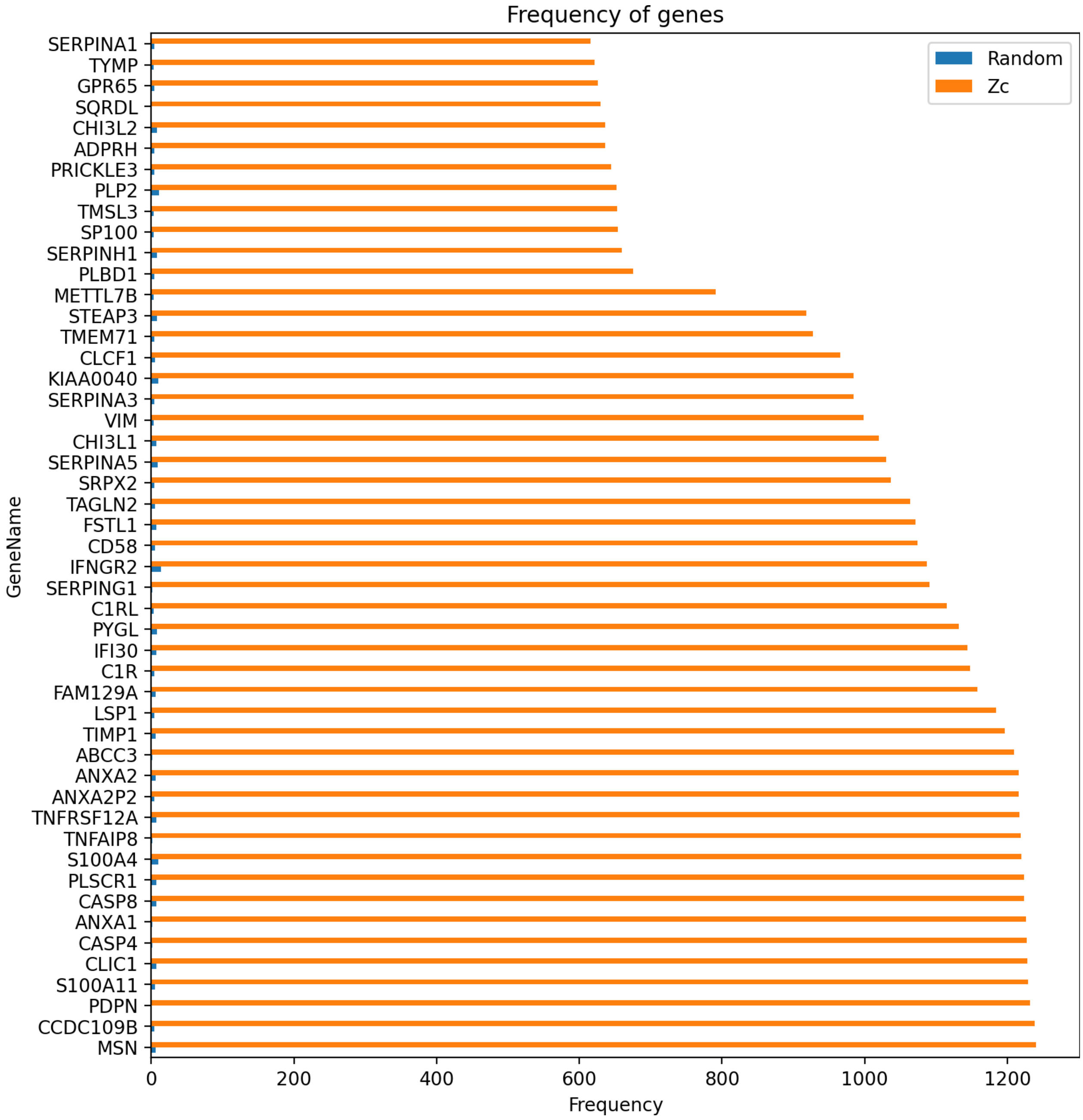

Frequency of genes in all random gene sets vs. scores of the fixed-point gene set of GBMLGG

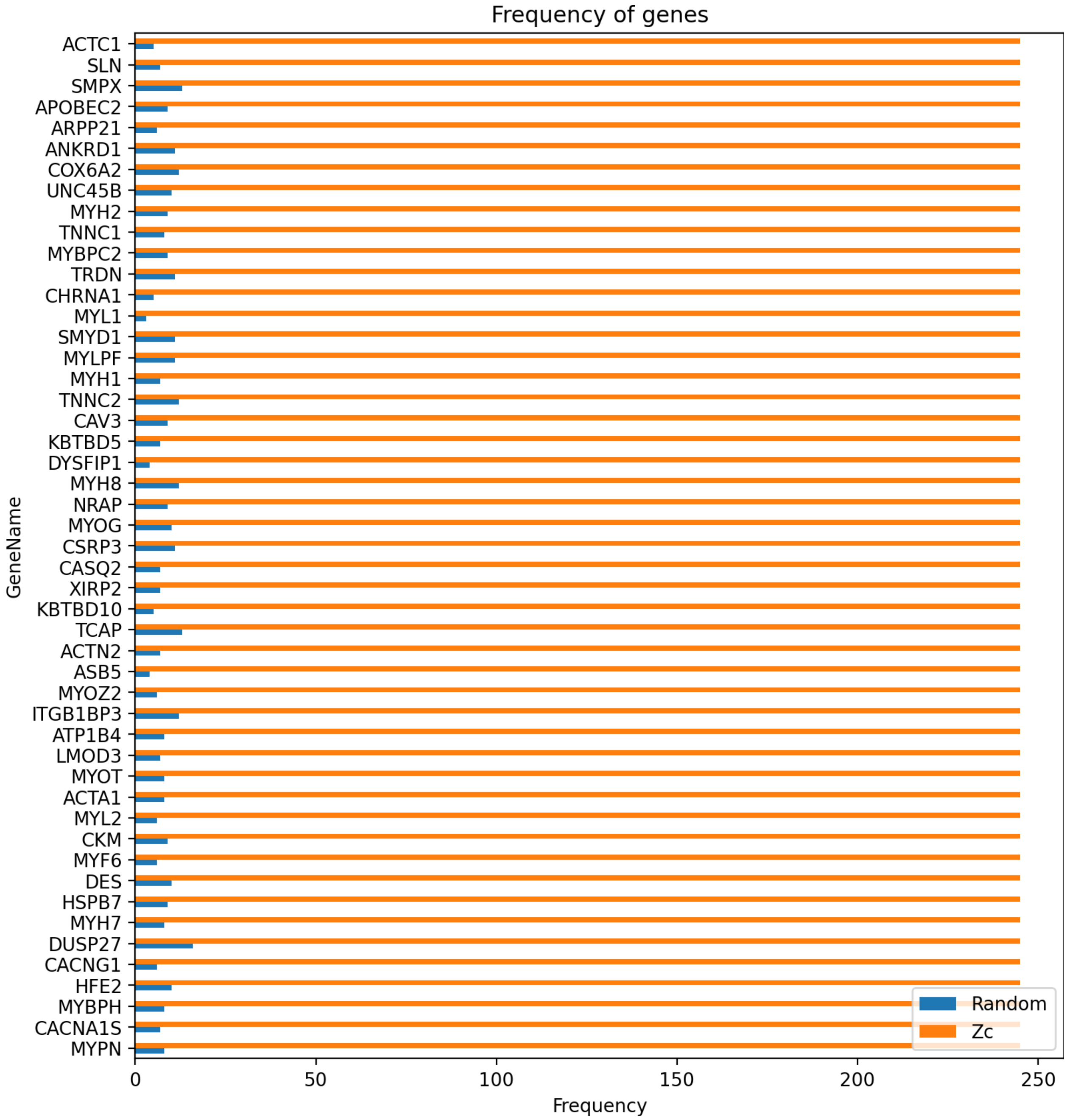

Frequency of genes in all random gene sets vs. scores of the fixed-point gene set of HNSC

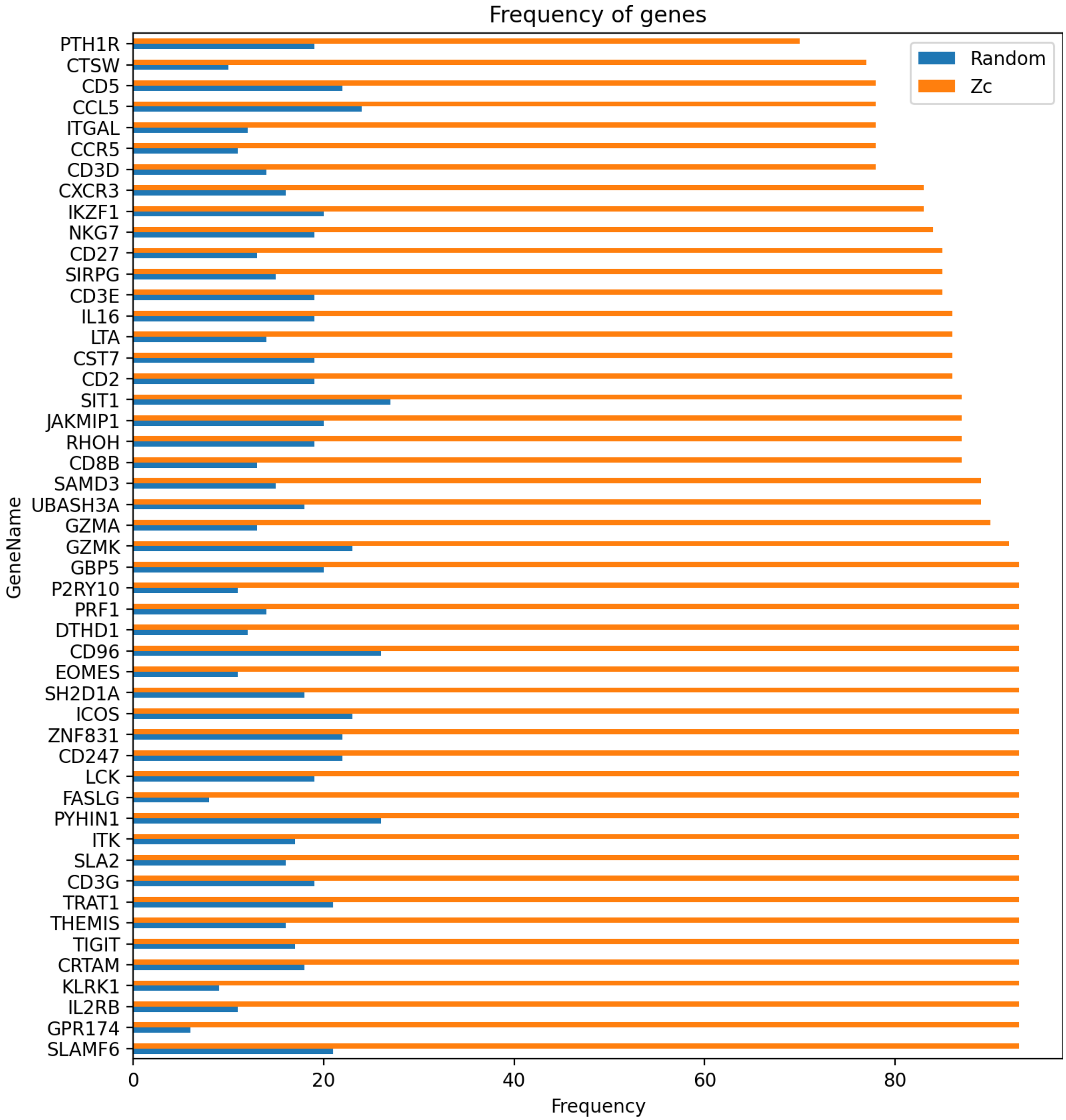

Frequency of genes in all random gene sets vs. scores of the fixed-point gene set of KIPAN

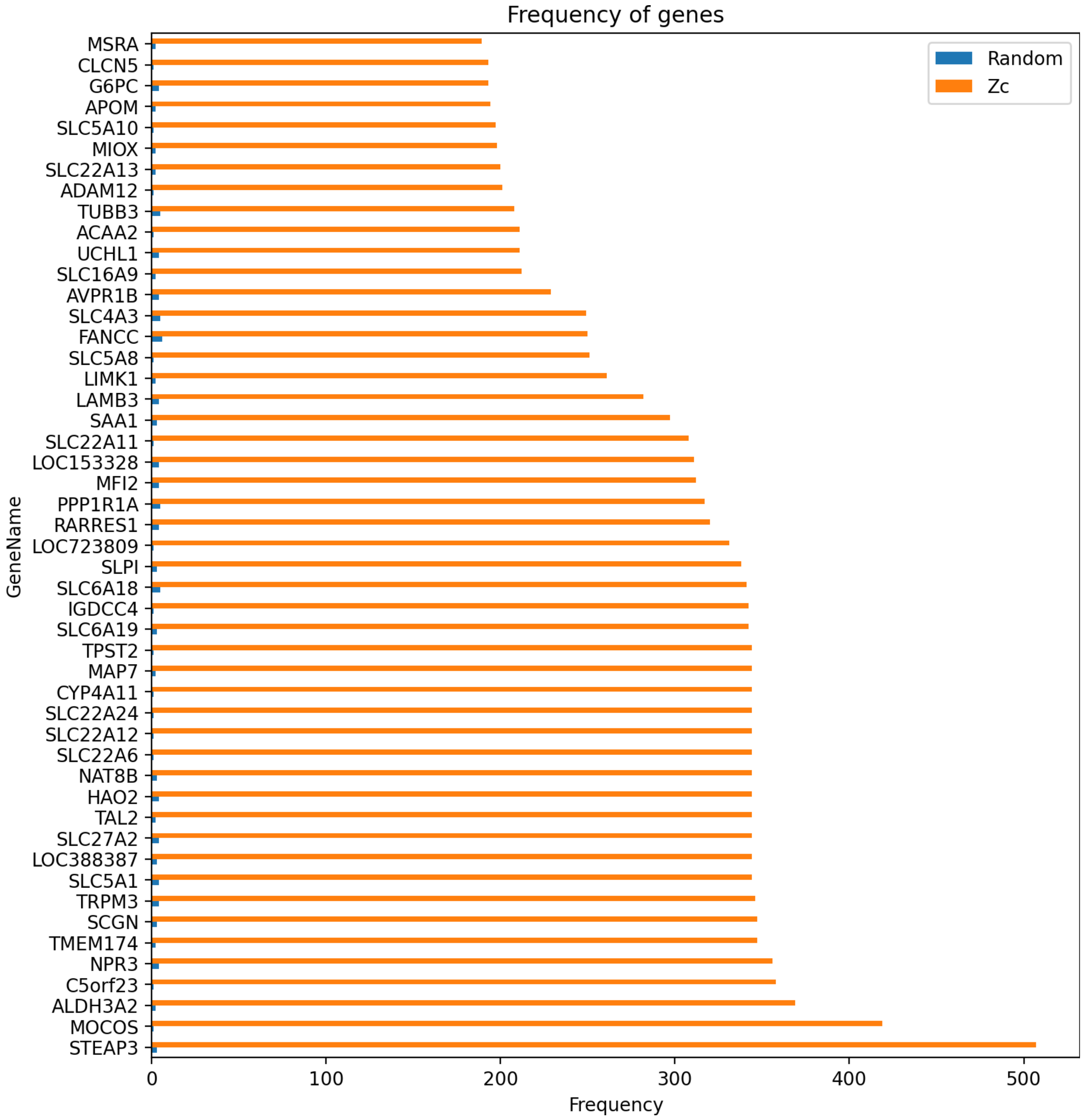

Frequency of genes in all random gene sets vs. scores of the fixed-point gene set of KIRC

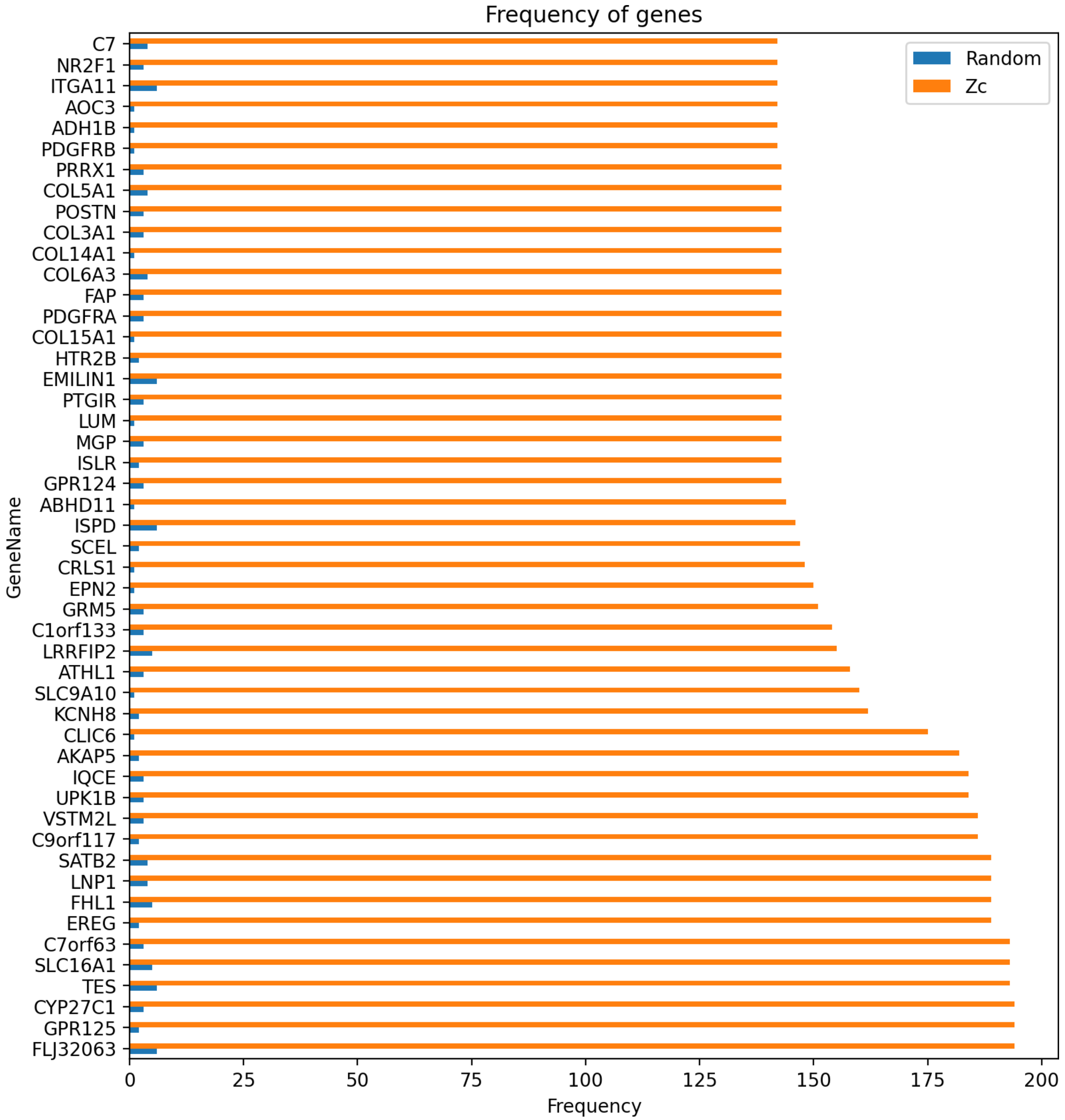

Frequency of genes in all random gene sets vs. scores of the fixed-point gene set of KIRP

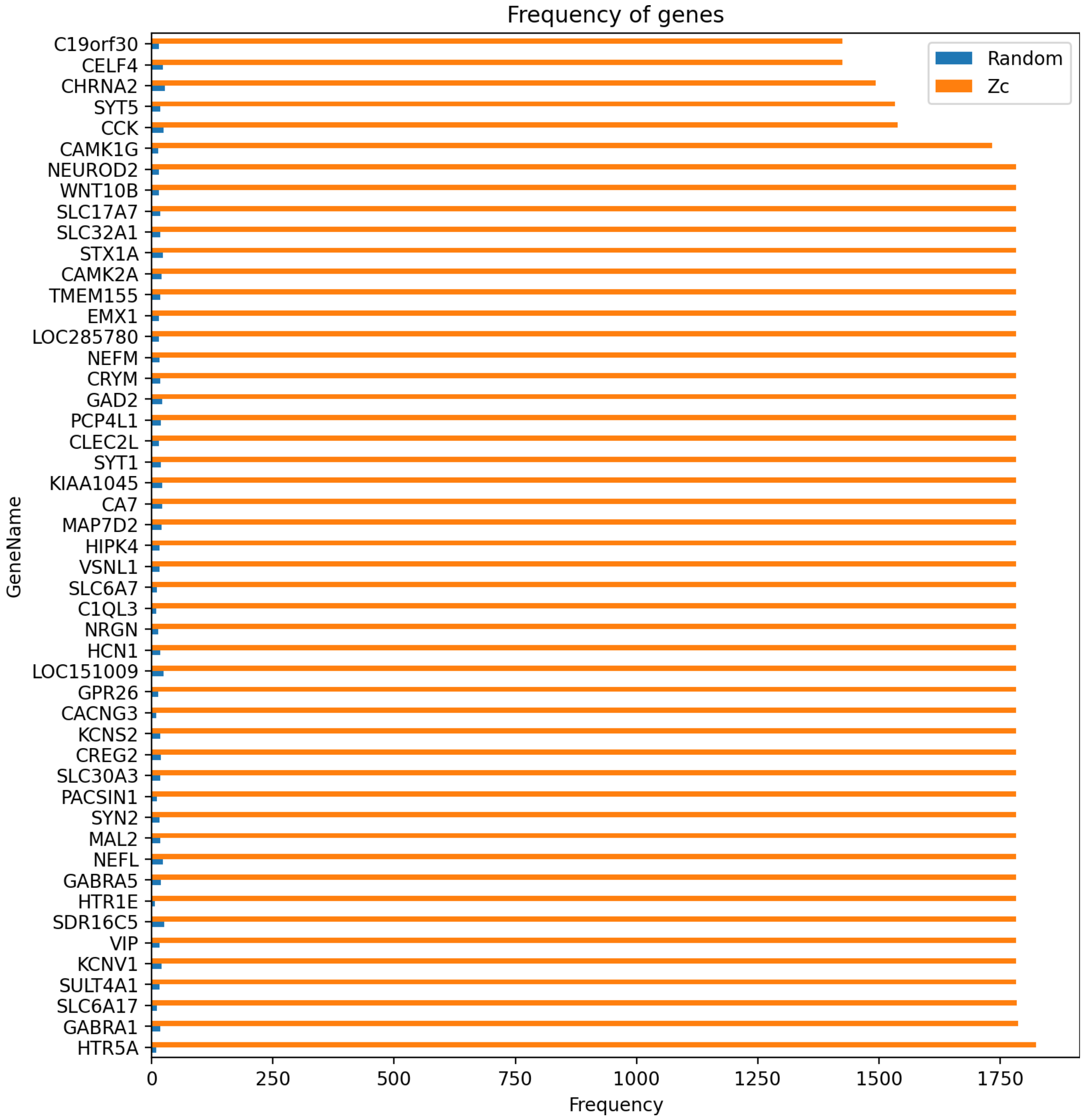

Frequency of genes in all random gene sets vs. scores of the fixed-point gene set of LGG

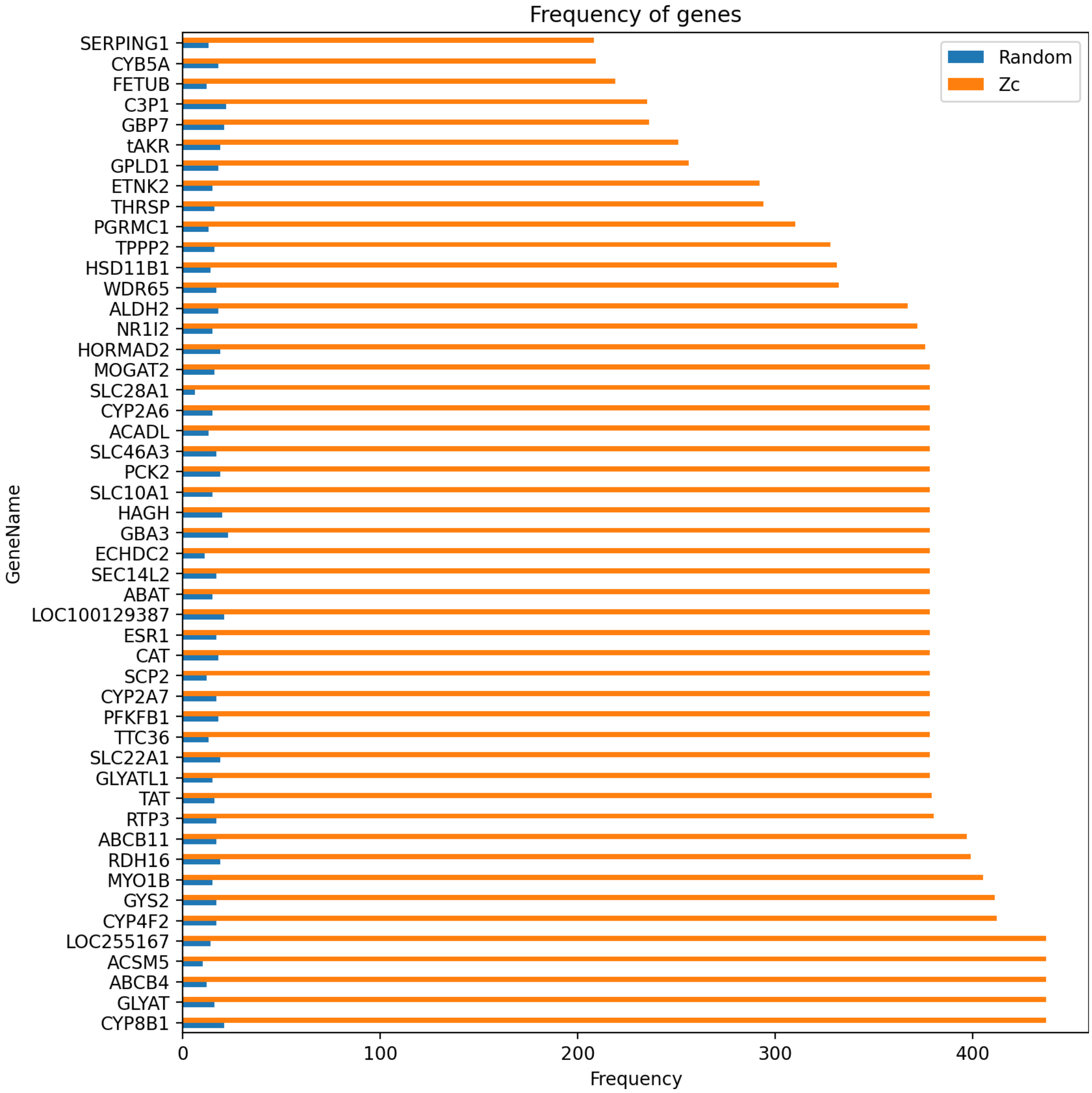

Frequency of genes in all random gene sets vs. scores of the fixed-point gene set of LIHC

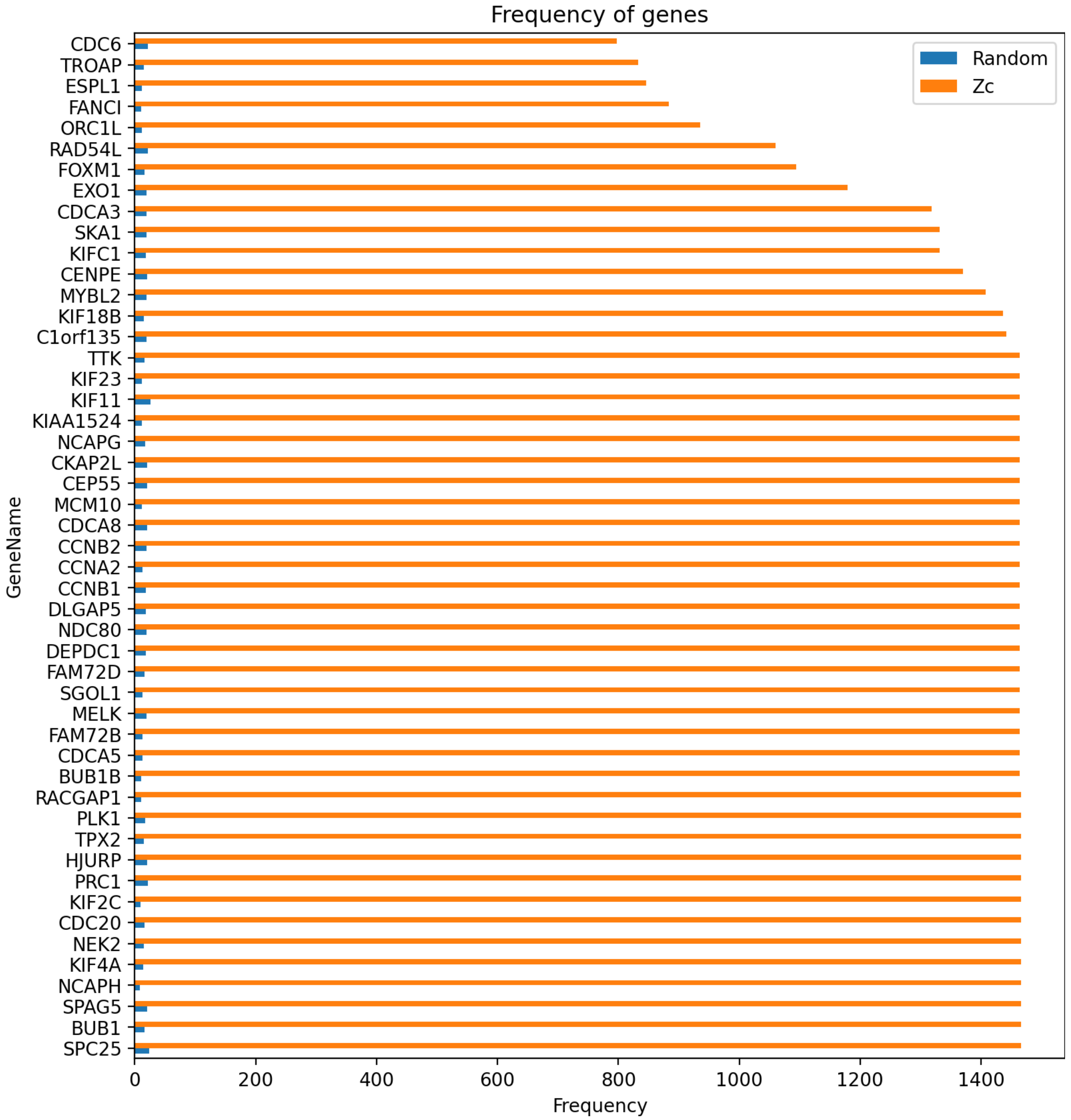

Frequency of genes in all random gene sets vs. scores of the fixed-point gene set of LUAD

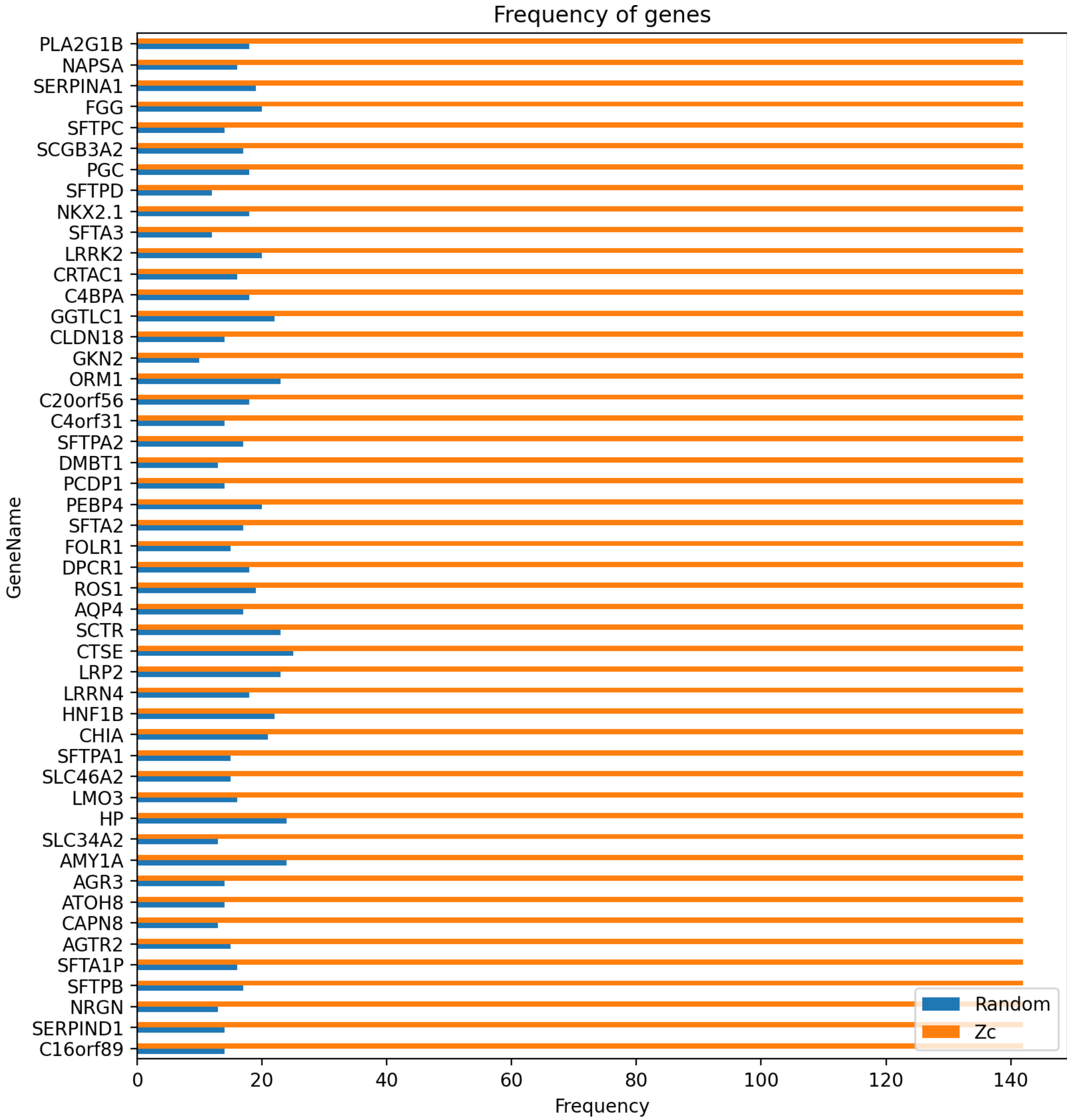

Frequency of genes in all random gene sets vs. scores of the fixed-point gene set of LUSC

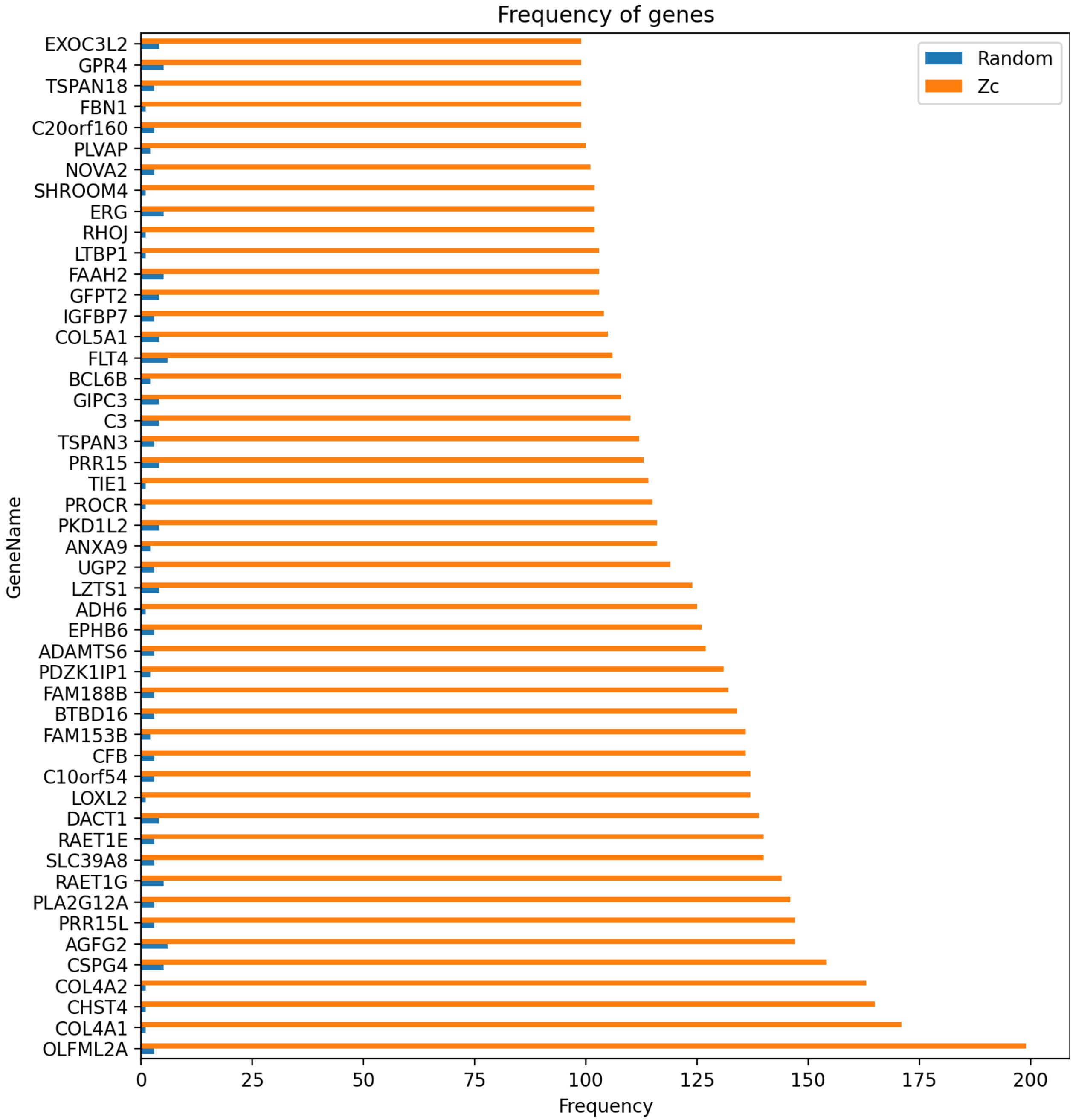

Frequency of genes in all random gene sets vs. scores of the fixed-point gene set of MESO

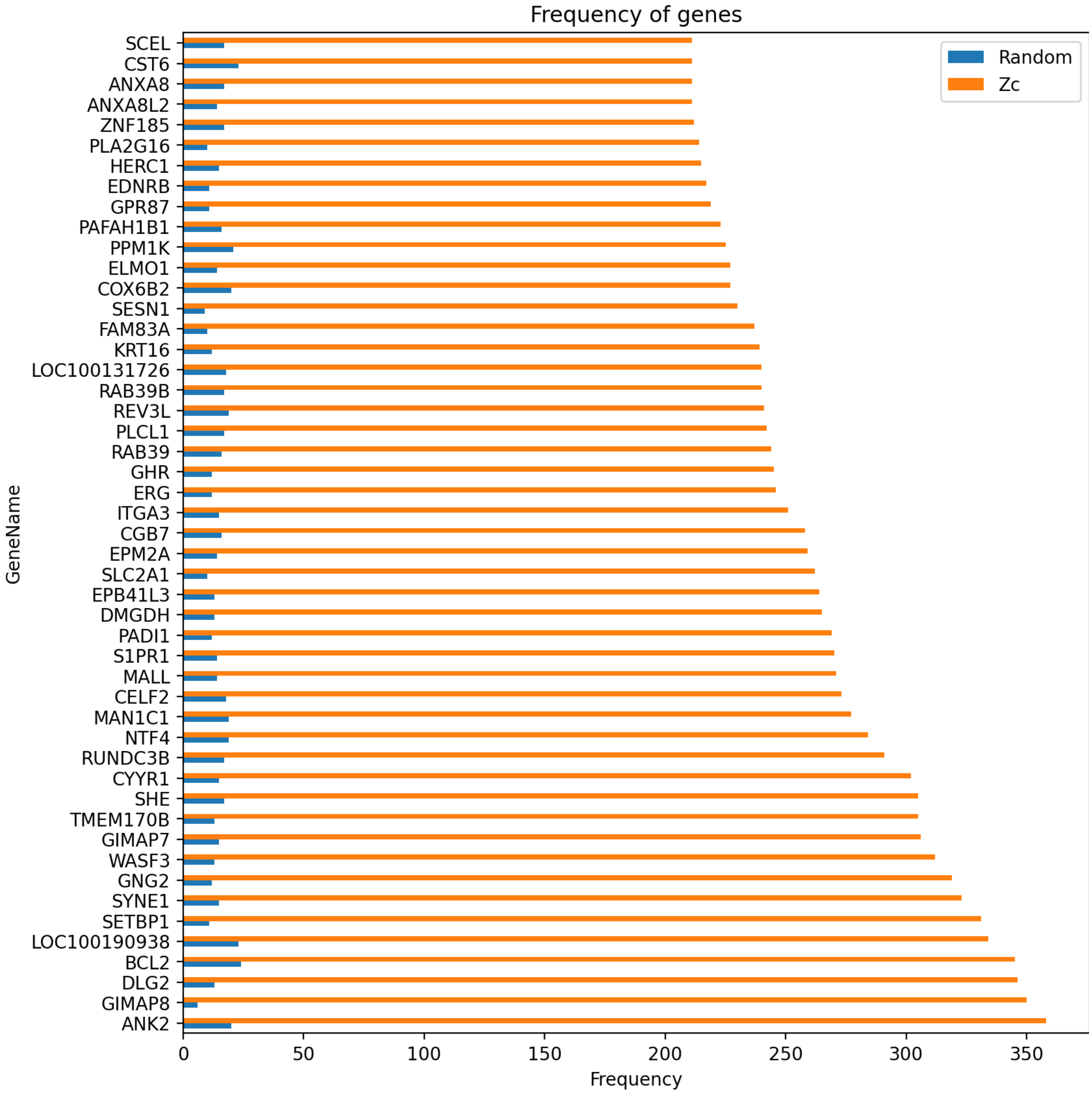

Frequency of genes in all random gene sets vs. scores of the fixed-point gene set OF PAAD

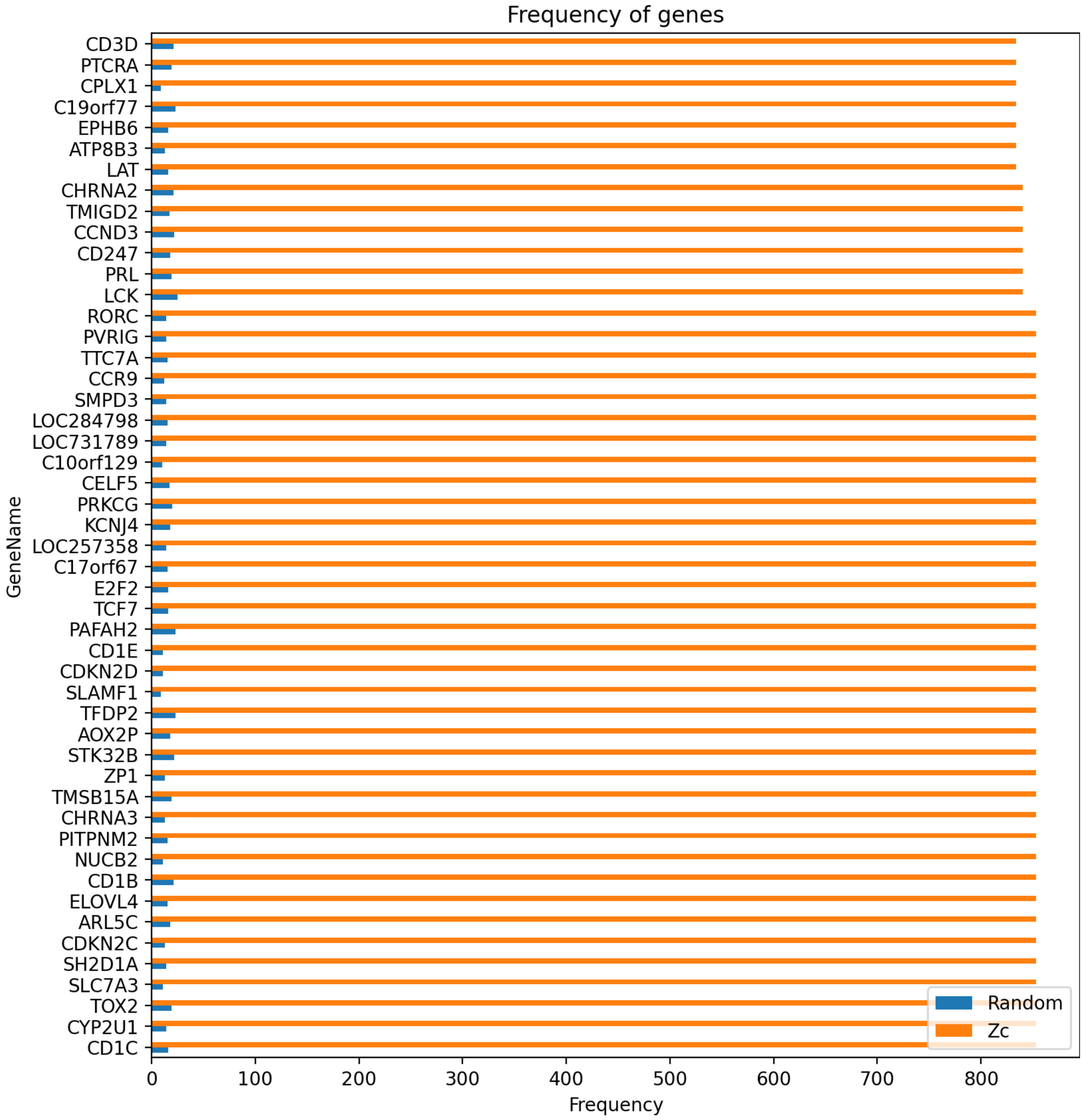

Frequency of genes in all random gene sets vs. scores of the fixed-point gene set of THYM

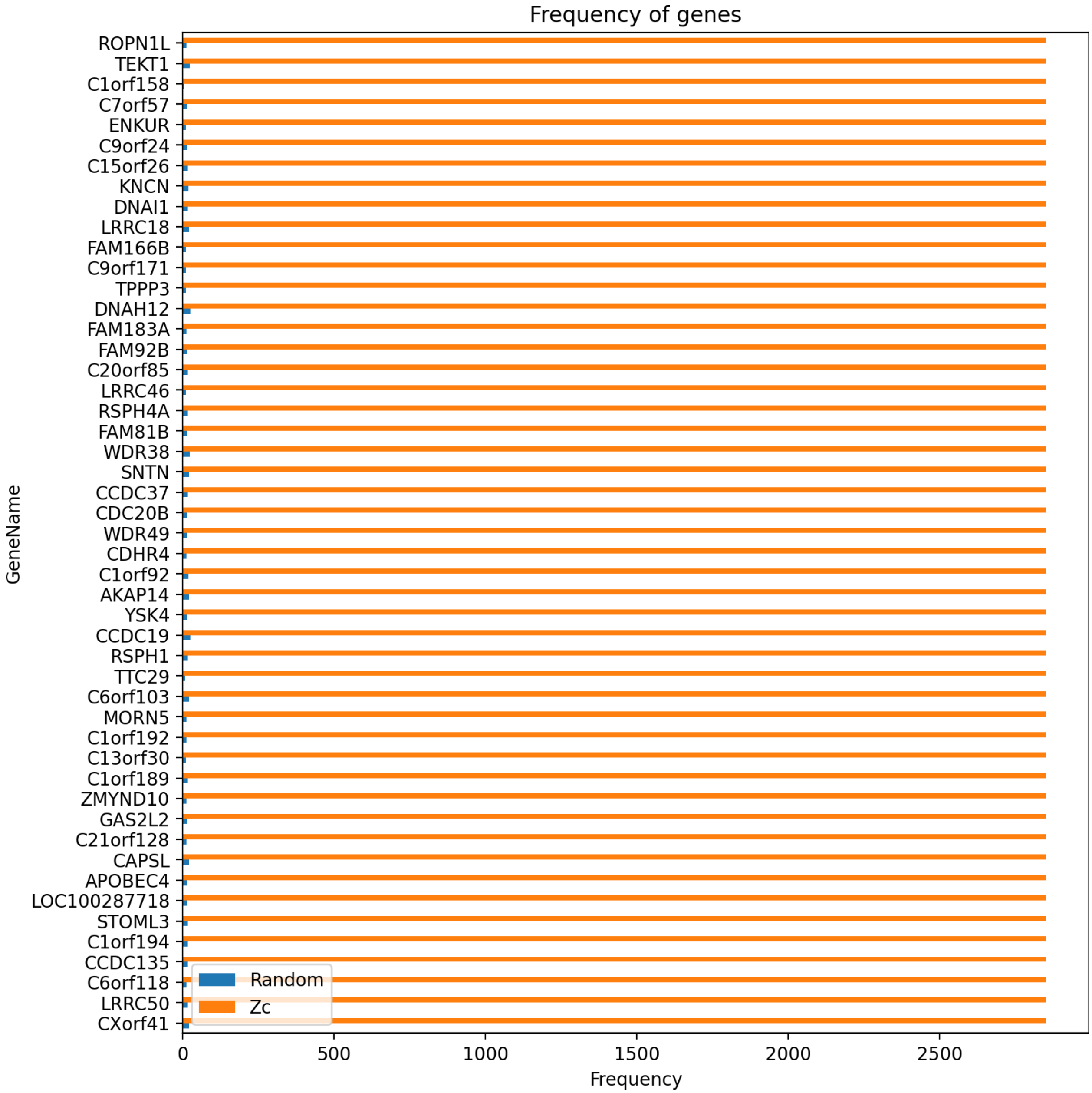

Frequency of genes in all random gene sets vs. scores of the fixed-point gene set of UCEC

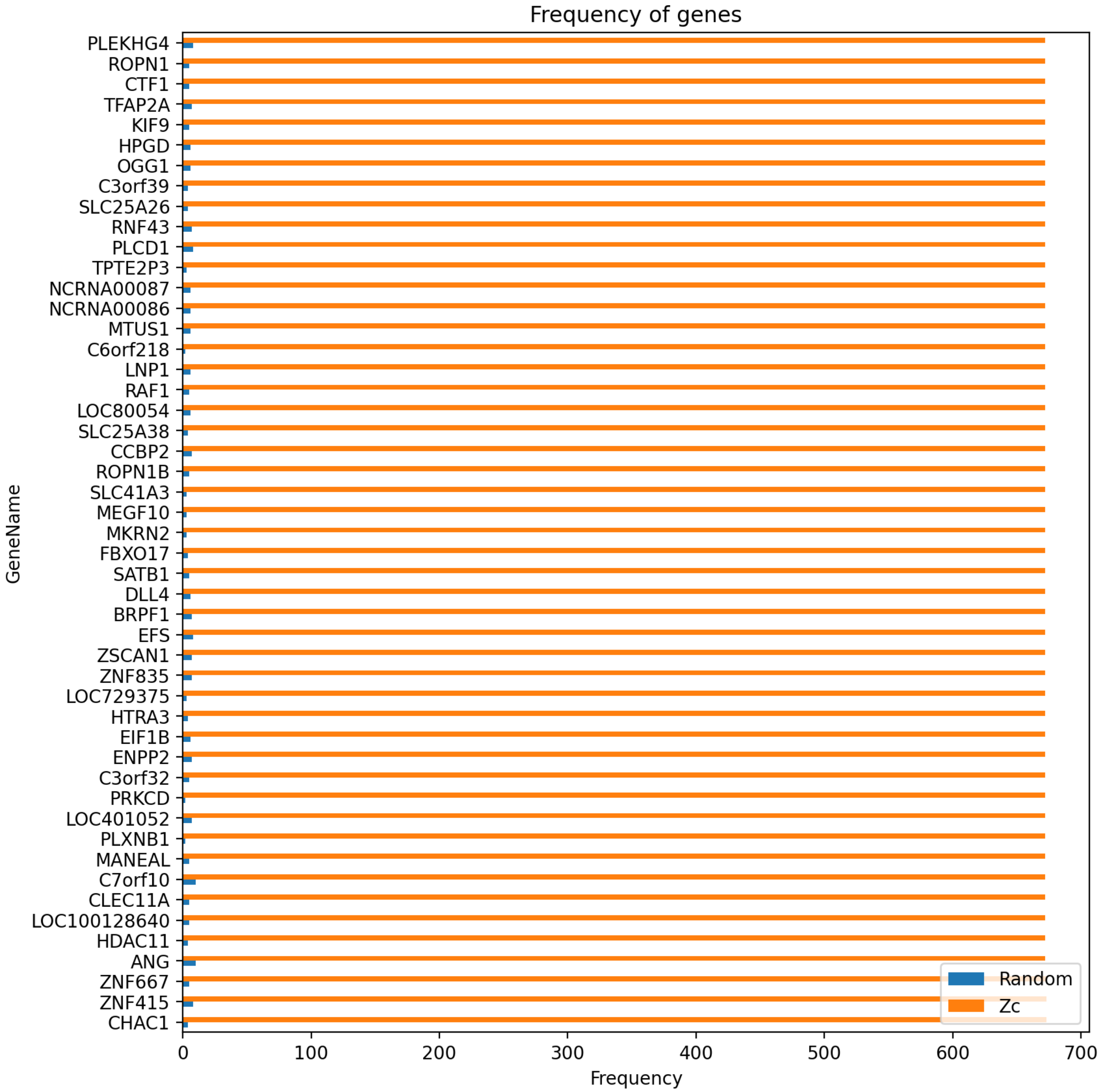

Frequency of genes in all random gene sets vs. scores of the fixed-point gene set of UVM
