# Supplementary material for: Removing the association of random gene sets and survival time in cancers with positive random bias using fixed-point gene set
Source: Sci Rep. 2023 May 29;13:8663. doi: 10.1038/s41598-023-35588-5 (PMC10226989; doi:10.1038/s41598-023-35588-5)
Supplement: Supplementary file 5 — Supplementary Information 2. [file 41598_2023_35588_MOESM5_ESM.pdf]

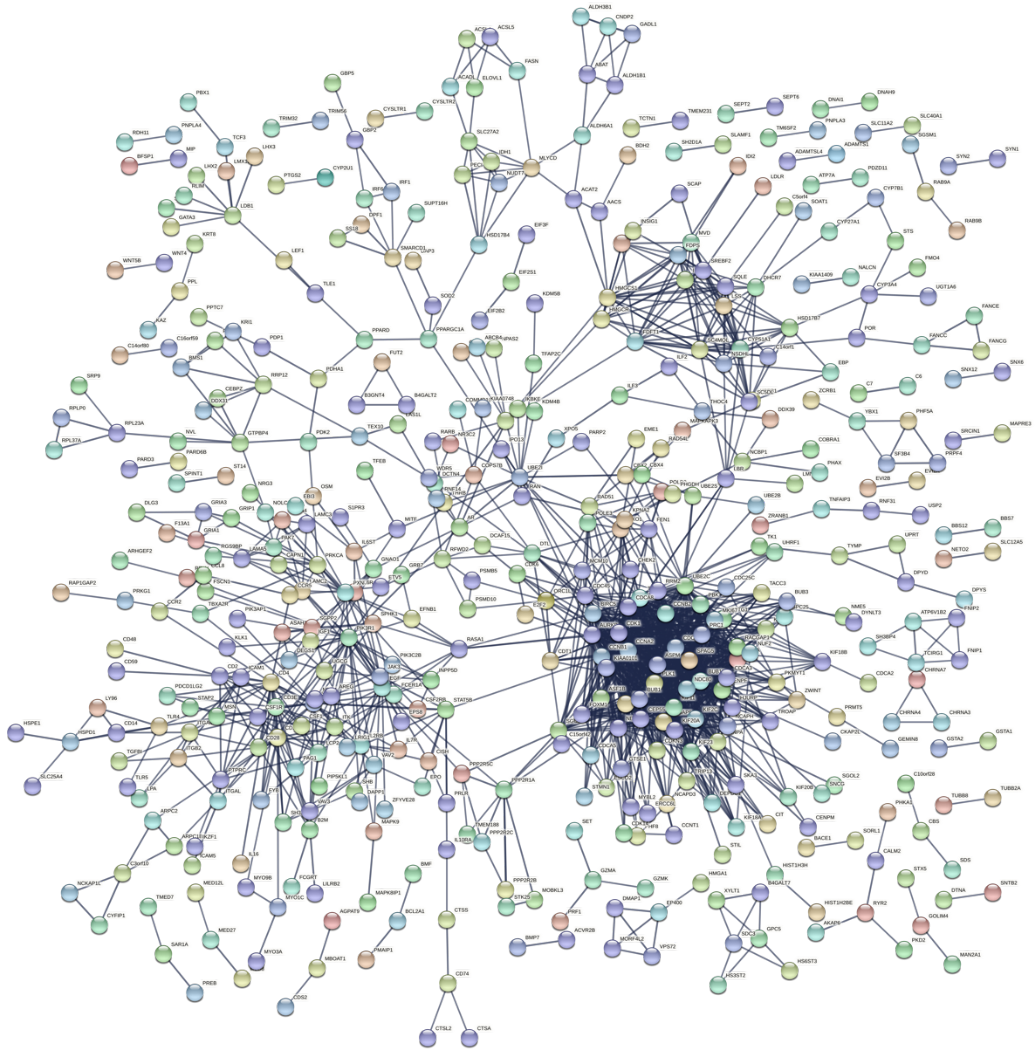

Protein-protein interaction network of fixed-point set of ACC. Figure were constructed using the STRING database (version 11.5; https://string-db.org/).

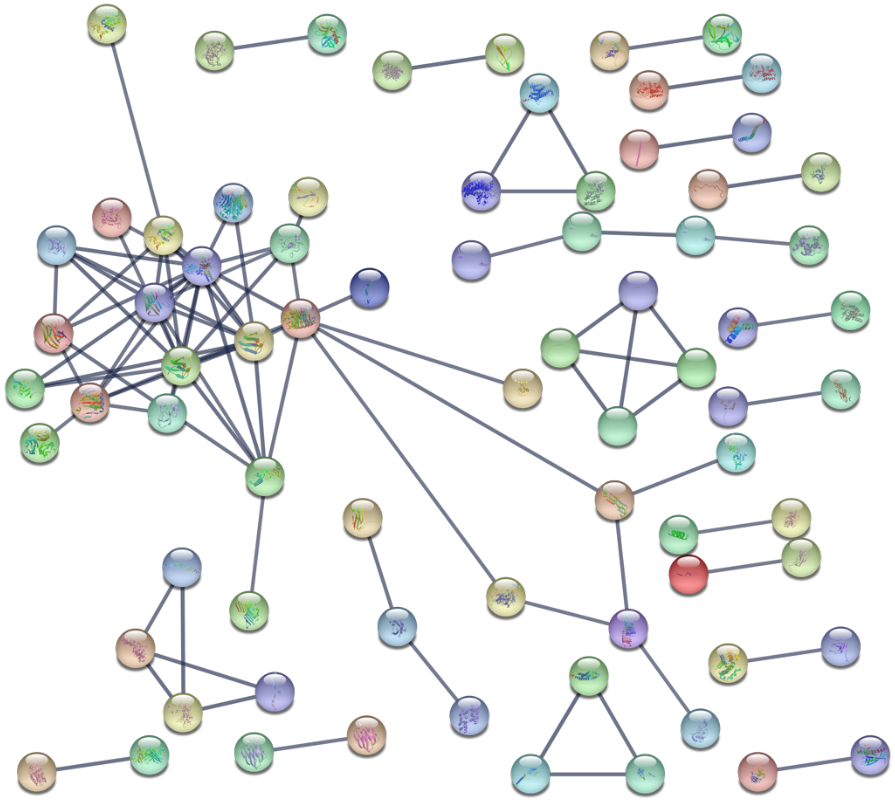

Protein-protein interaction network of fixed-point set of BLCA. Figure were constructed using the STRING database (version 11.5; https://string-db.org/).

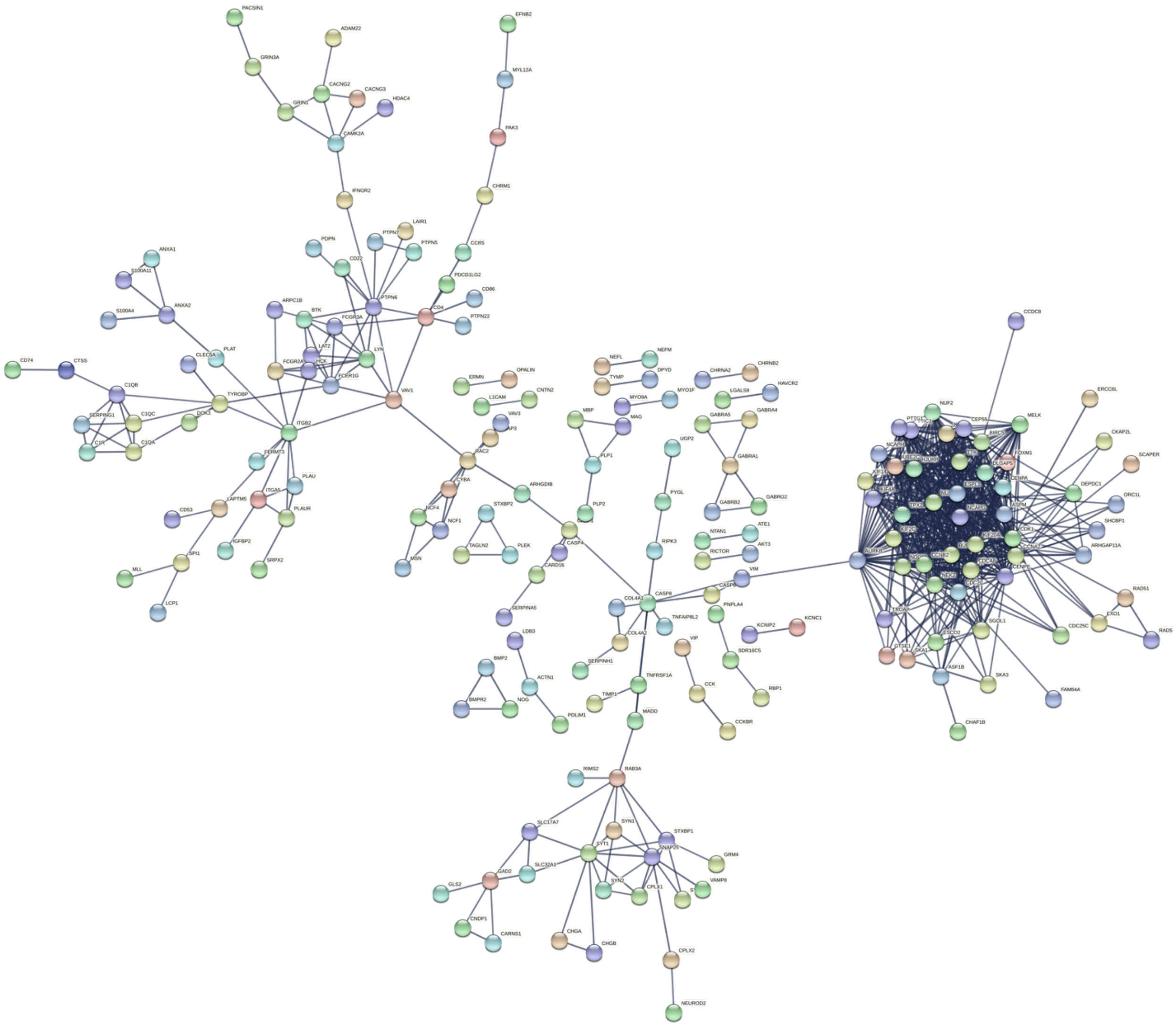

Protein-protein interaction network of fixed-point set of GBMLGG. Figure were constructed using the STRING database (version 11.5; https://string-db.org/).

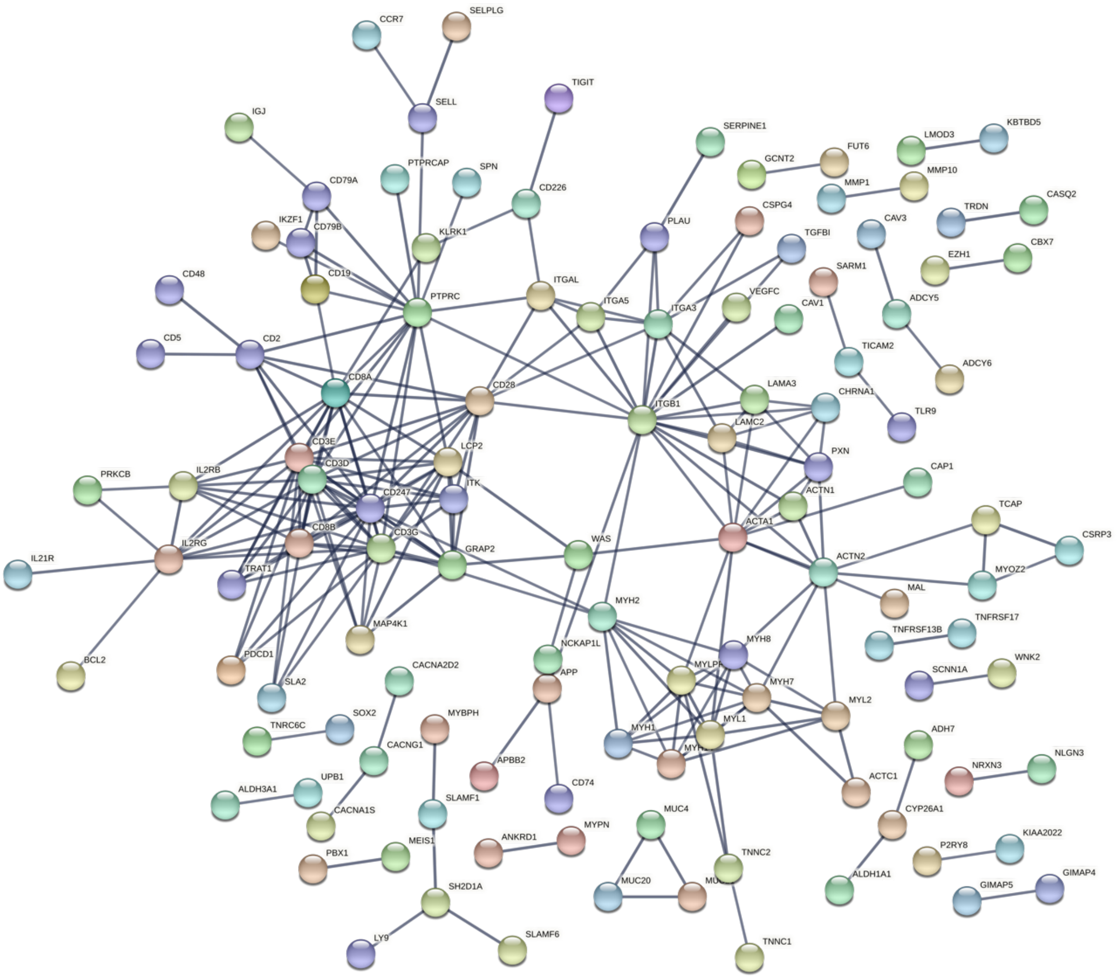

Protein-protein interaction network of fixed-point set of HNSC. Figure were constructed using the STRING database (version 11.5; https://string-db.org/).

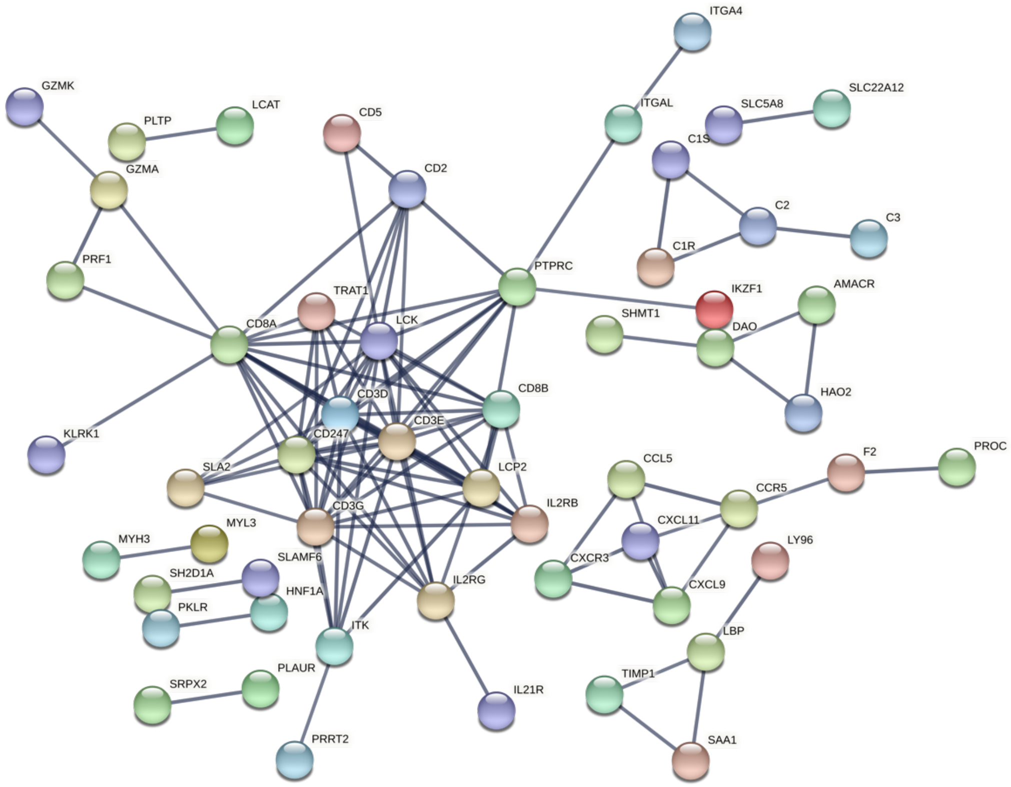

Protein-protein interaction network of fixed-point set of KIPAN. Figure were constructed using the STRING database (version 11.5; https://string-db.org/).

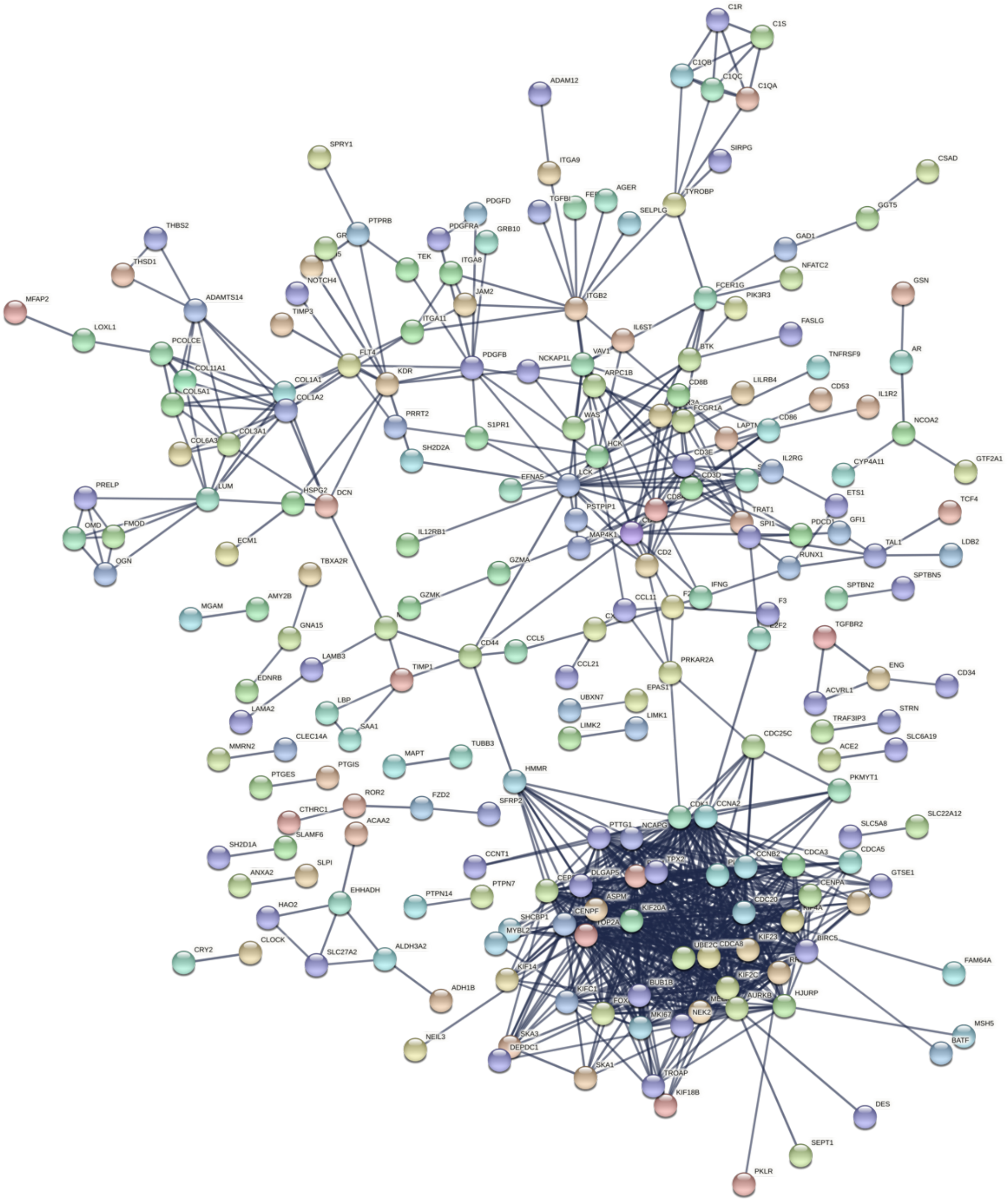

Protein-protein interaction network of fixed-point set of KIRC. Figure were constructed using the STRING database (version 11.5; https://string-db.org/).

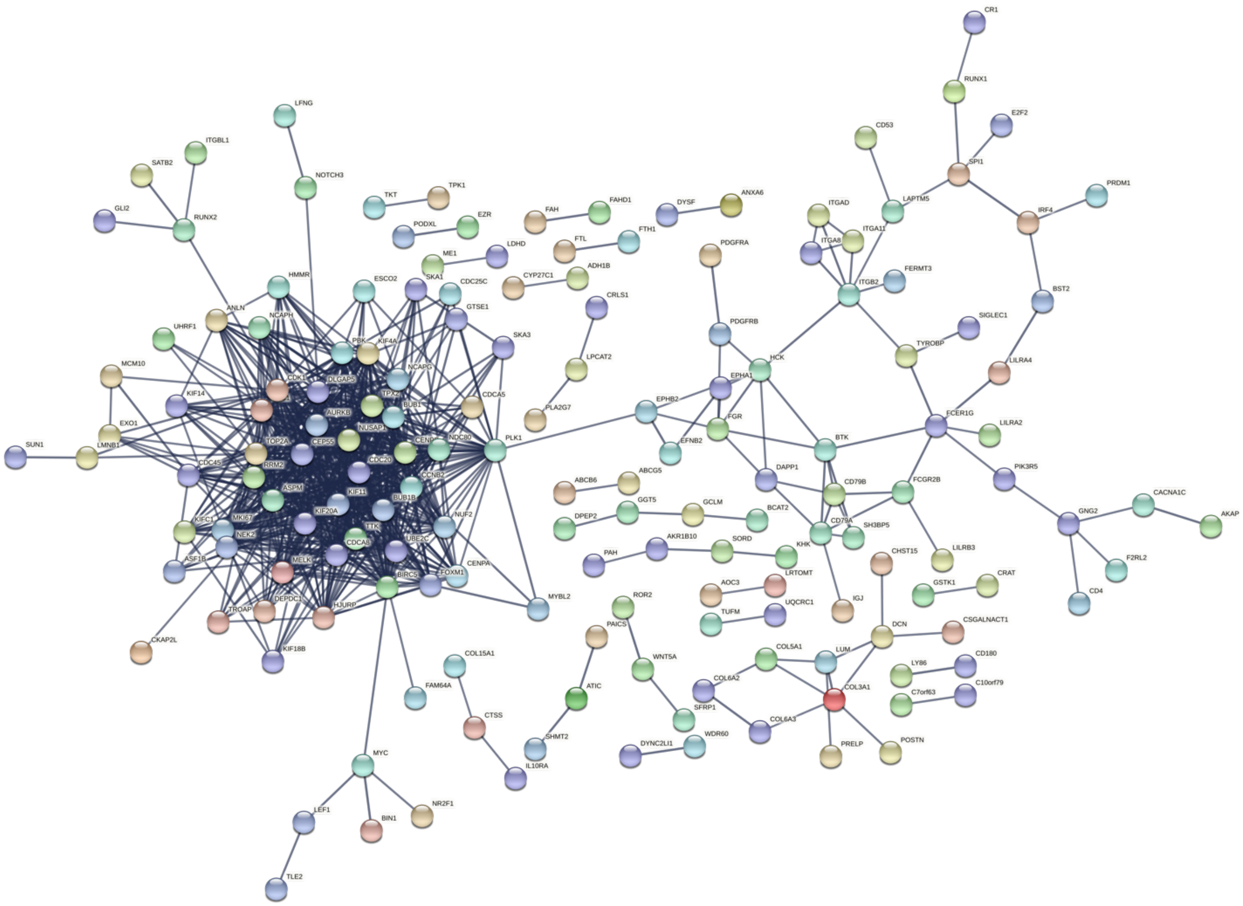

Protein-protein interaction network of fixed-point set of KIRP. Figure were constructed using the STRING database (version 11.5; https://string-db.org/).

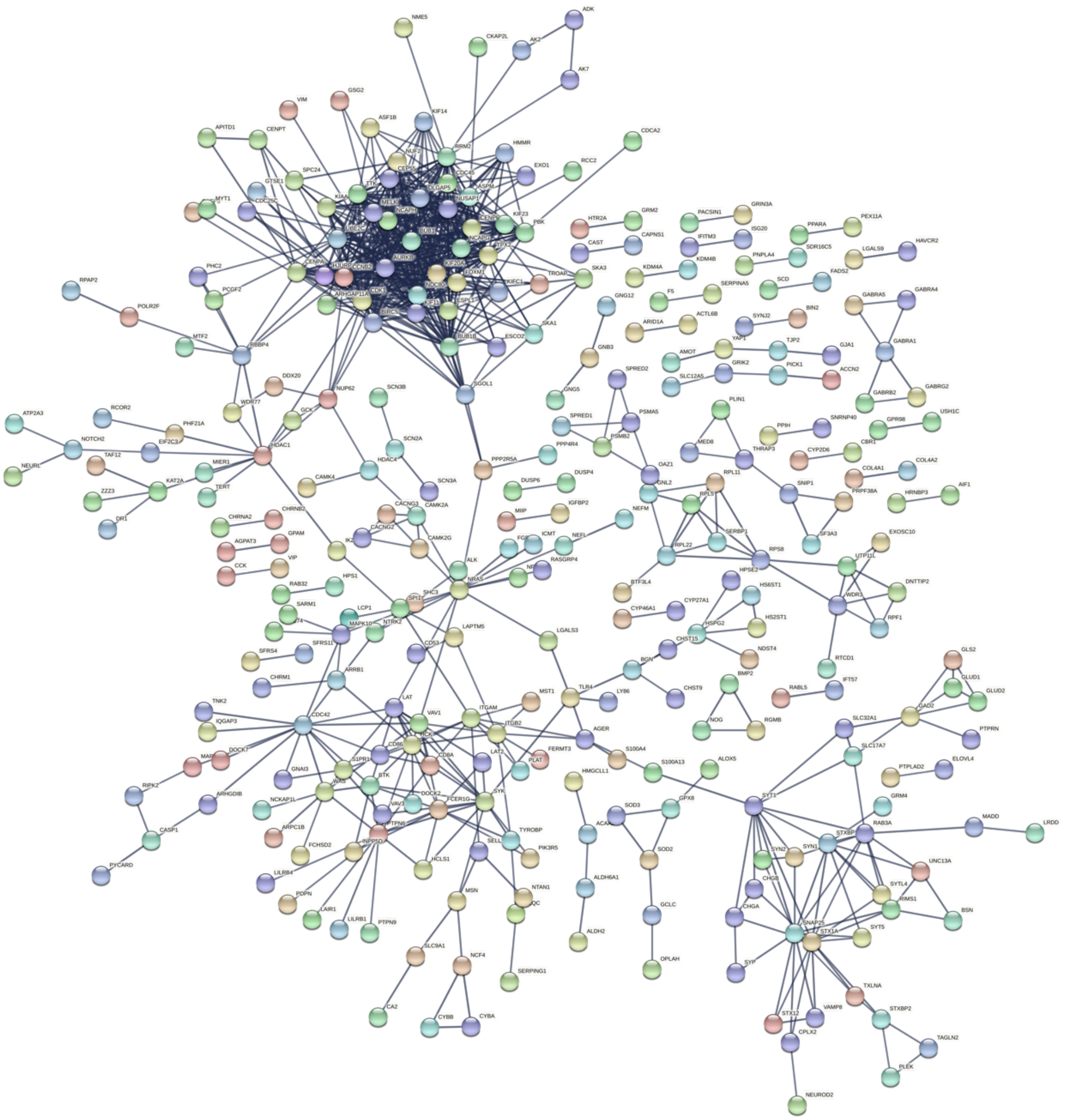

Protein-protein interaction network of fixed-point set of LGG. Figure were constructed using the STRING database (version 11.5; https://string-db.org/).

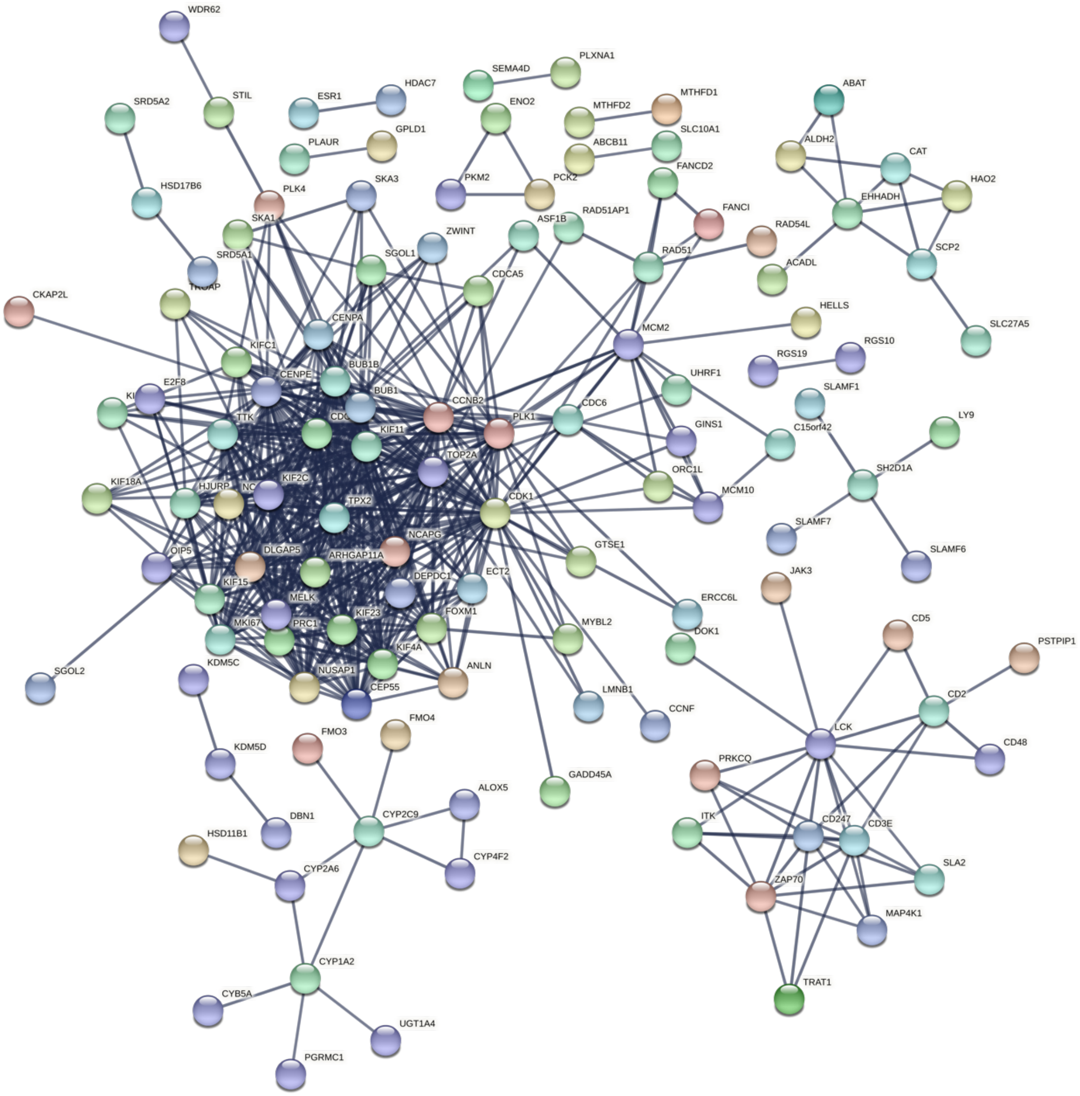

Protein-protein interaction network of fixed-point set of LIHC. Figure were constructed using the STRING database (version 11.5; https://string-db.org/).

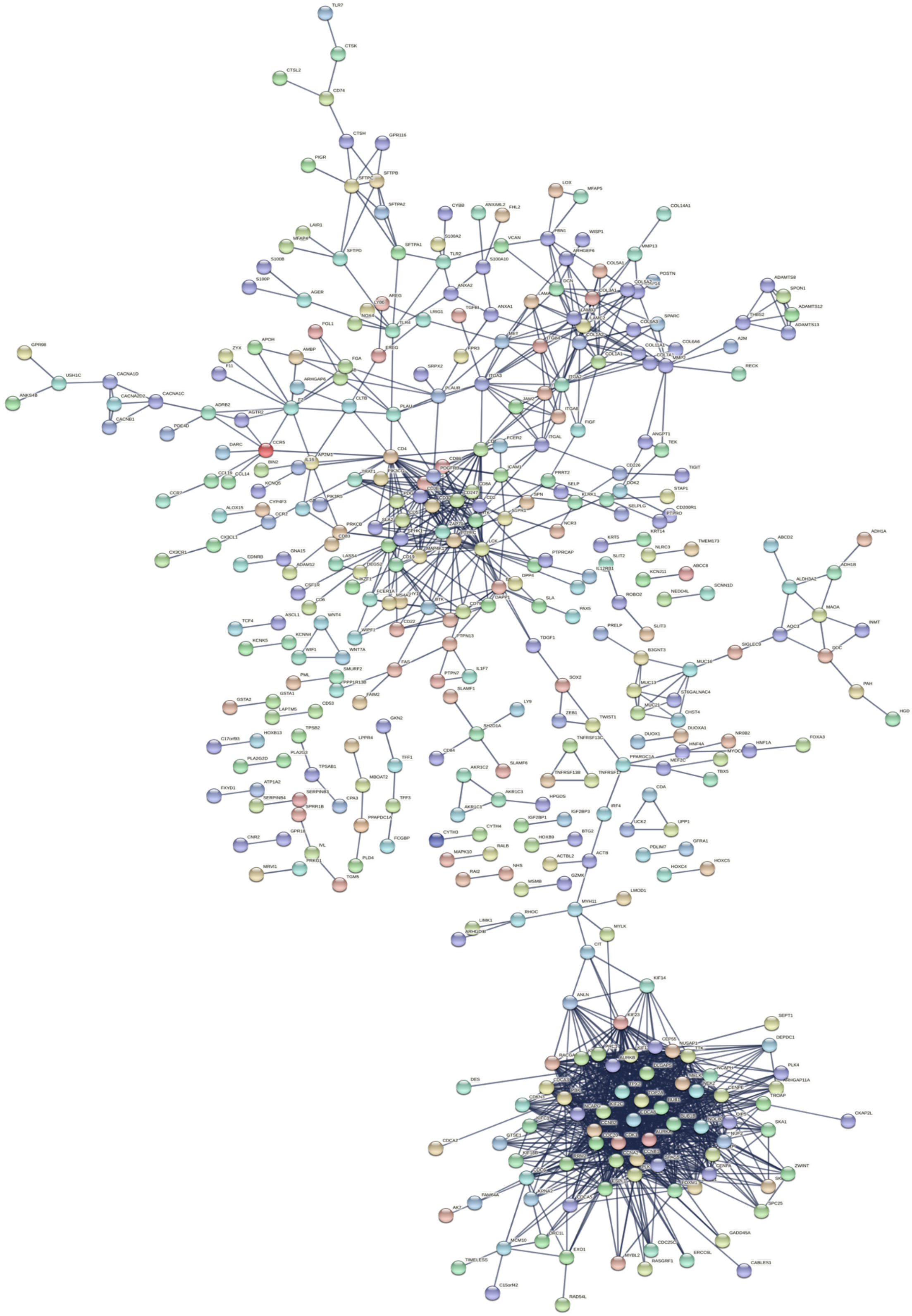

Protein-protein interaction network of fixed-point set of LUAD. Figure were constructed using the STRING database (version 11.5; https://string-db.org/).

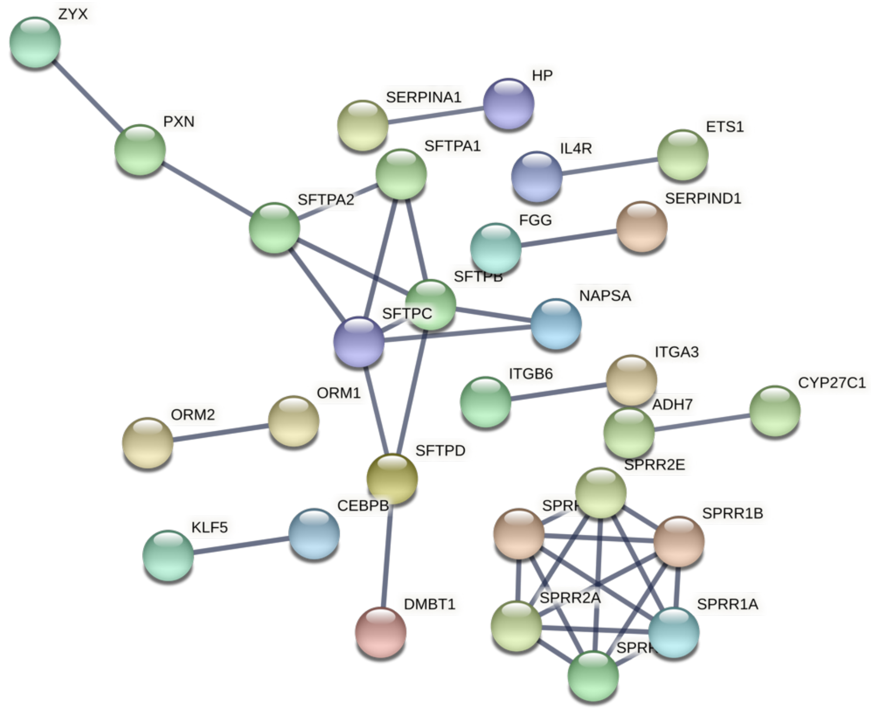

Protein-protein interaction network of fixed-point set of LUSC. Figure were constructed using the STRING database (version 11.5; https://string-db.org/).

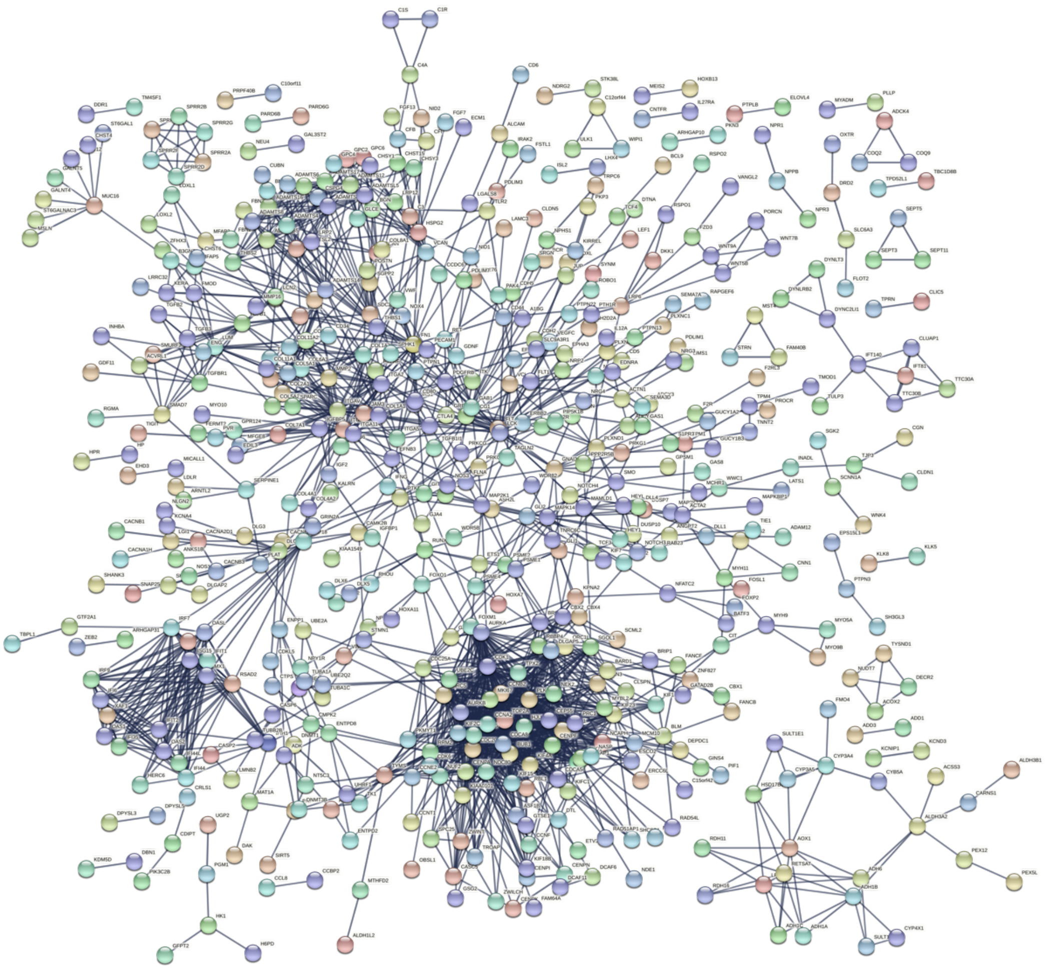

Protein-protein interaction network of fixed-point set of MESO. Figure were constructed using the STRING database (version 11.5; https://string-db.org/).

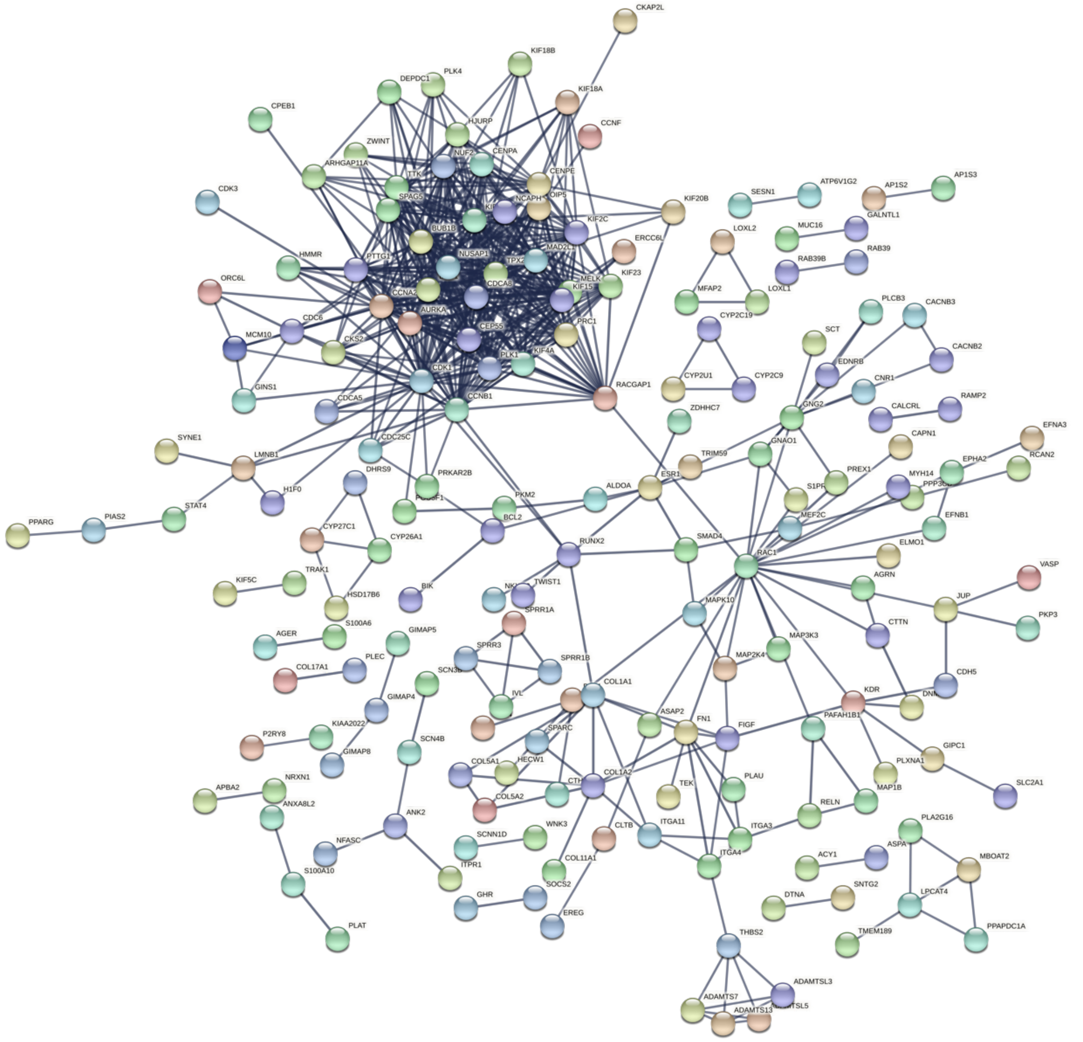

Protein-protein interaction network of fixed-point set of PAAD. Figure were constructed using the STRING database (version 11.5; https://string-db.org/).

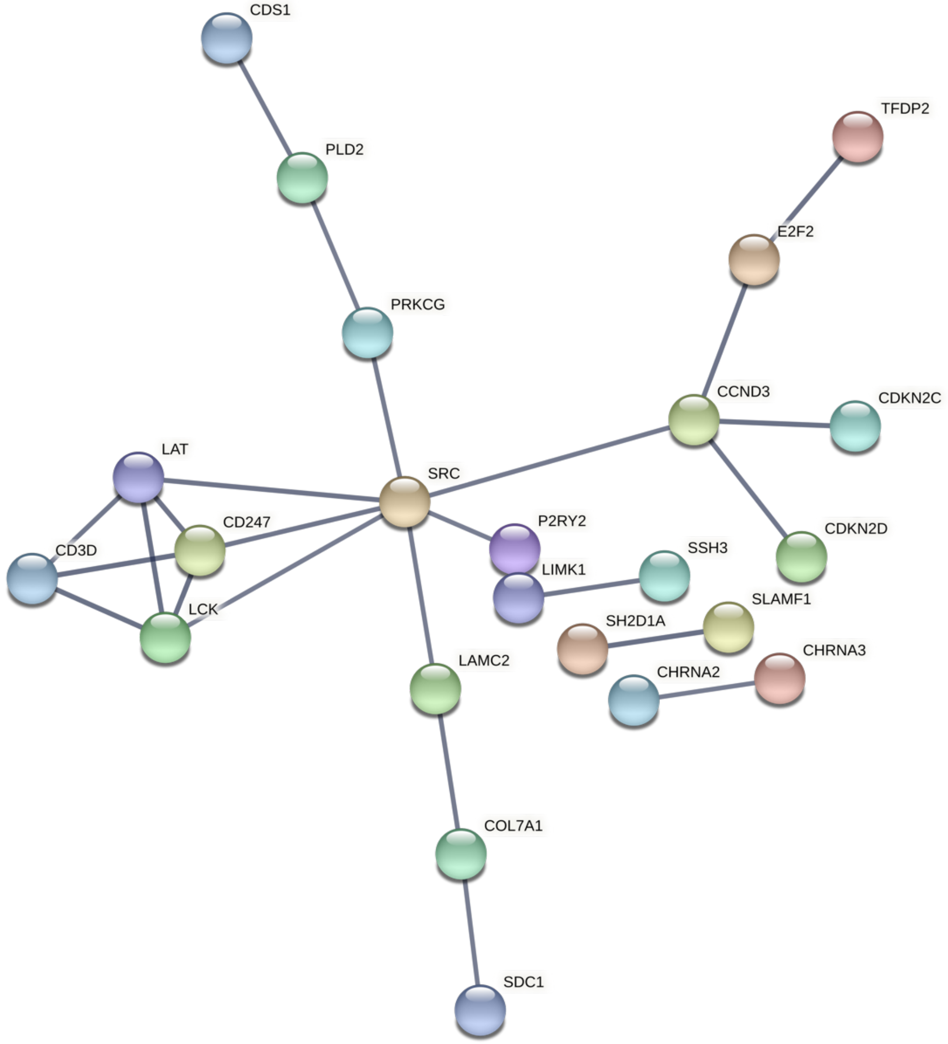

Protein-protein interaction network of fixed-point set of THYM. Figure were constructed using the STRING database (version 11.5; https://string-db.org/).

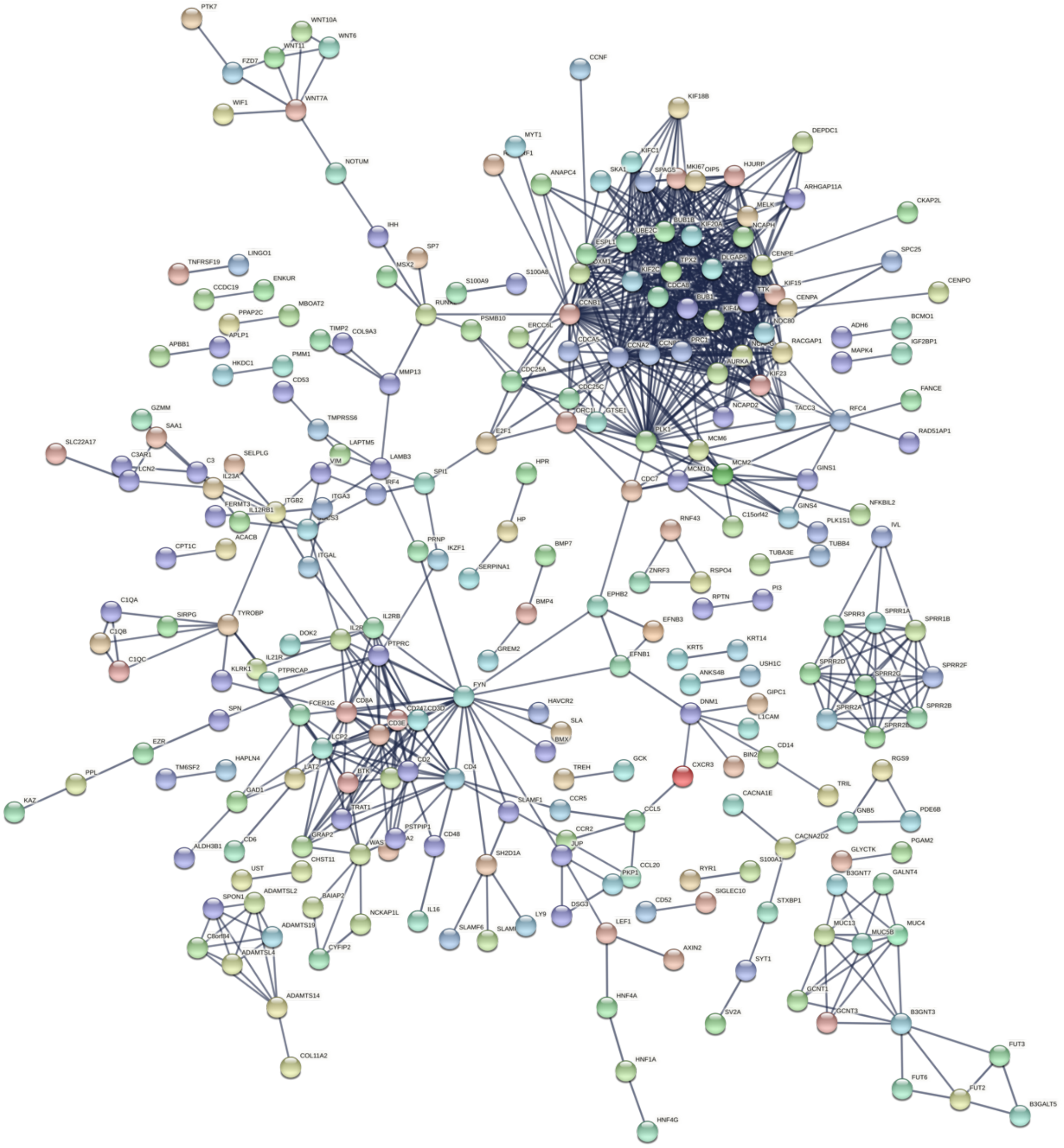

Protein-protein interaction network of fixed-point set of UCEC. Figure were constructed using the STRING database (version 11.5; https://string-db.org/).

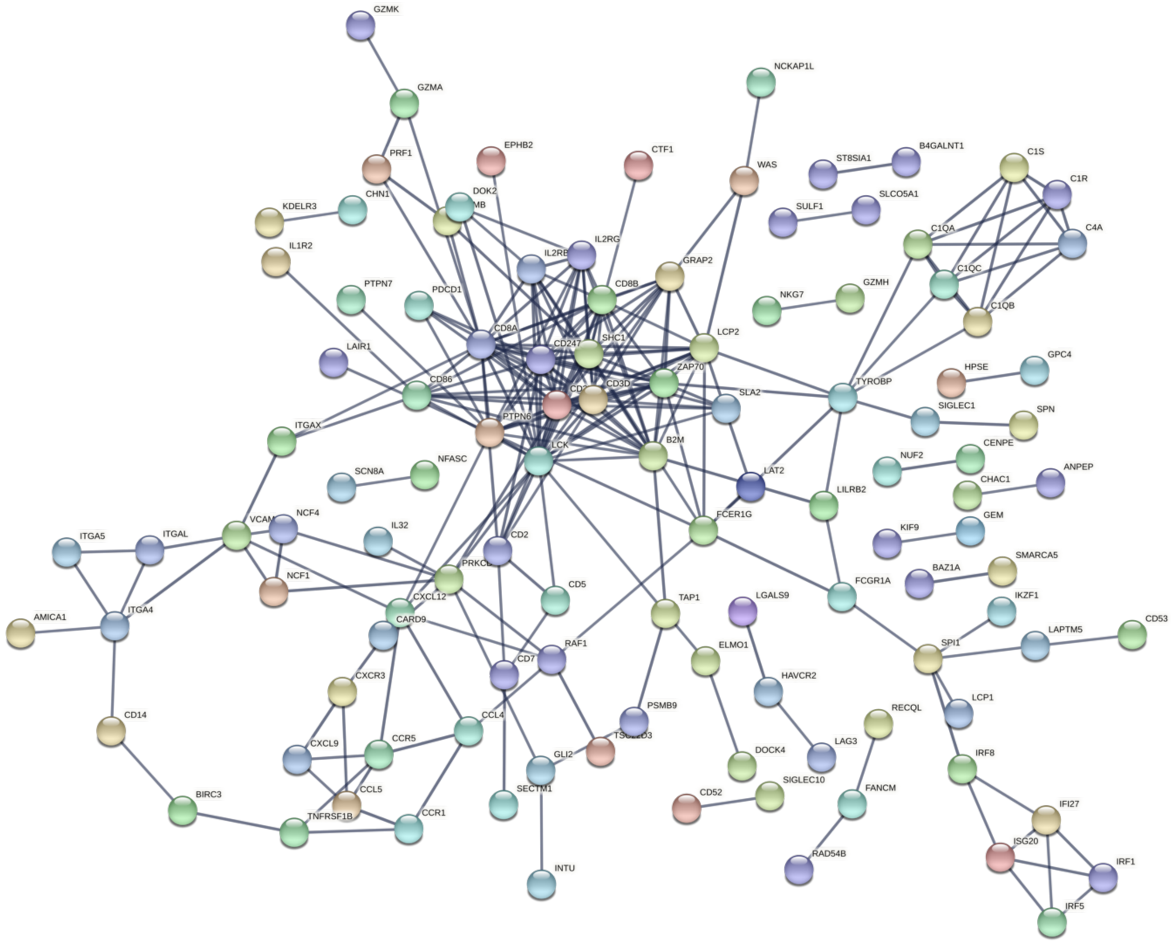

Protein-protein interaction network of fixed-point set of UVM. Figure were constructed using the STRING database (version 11.5; https://string-db.org/).
