# Supplementary material for: Removing the association of random gene sets and survival time in cancers with positive random bias using fixed-point gene set
Source: Sci Rep. 2023 May 29;13:8663. doi: 10.1038/s41598-023-35588-5 (PMC10226989; doi:10.1038/s41598-023-35588-5)
Supplement: Supplementary file 6 — Supplementary Information 3. [file 41598_2023_35588_MOESM6_ESM.pdf]

Cancer	AVG-DIS-CLS	Z_C-DIS
**ACC**	0.0318	0.001
**BLCA**	0.0104	0.016
**BRCA**	0.0246	0.002
**GBMLGG**	0.1544	0.207
**HNSC**	0.0231	0.154
**KIPAN**	0.2858	0.227
**KIRC**	0.0839	0.21
**KIRP**	0.2190	0.004
**LGG**	0.1072	0.141
**LIHC**	0.0585	0.025
**LUAD**	0.1717	0.062
**LUSC**	0.0169	0.010
**MESO**	0.0498	0.090
**PAAD**	0.0253	0.080
**THYM**	0.0416	0.020
**UCEC**	0.0274	0.270
**UVM**	0.0265	0.019

The names of 17 cancer types with positive random bias properties are listed in the first column of the table above. The average distance between each cluster proportion of the SSAR gene sets and 0.05 is displayed in the second column. For each cancer type, we used the following formula to determine this value:

$$\frac{\sum_{i=0}^{n} SSAR_{CL}(i) - 0.05}{n}$$

The second column represents the distance of proportion of SSAR gene sets after removing the effect of corresponding fixed-point set from 0.05.
