# Supplementary material for: Removing the association of random gene sets and survival time in cancers with positive random bias using fixed-point gene set
Source: Sci Rep. 2023 May 29;13:8663. doi: 10.1038/s41598-023-35588-5 (PMC10226989; doi:10.1038/s41598-023-35588-5)

Top ten genes of Z_C calculated by random gene sets of size 50

NO.	Gene	Score
1	C6orf97	397
2	KDM4B	397
3	MAPT	397
4	NXNL2	397
5	SPATA4	397
6	SPEF2	397
7	TPRG1	397
8	CASC1	396
9	PGR	396
10	SCUBE2	396

Top ten genes of Z_C calculated by random gene sets of size 100

NO.	Gene	Score
1	C6orf97	359
2	CASC1	359
3	KDM4B	359
4	MAPT	359
5	NXNL2	359
6	PGR	359
7	SCUBE2	359
8	SPATA4	340
9	SPEF2	340
10	TPRG1	340

Top ten genes of Z_C calculated by random gene sets of size 200

NO.	Gene	Score
1	C6orf97	425
2	CASC1	425
3	DNAH7	425
4	MAPT	425
5	GPR77	425
6	PGR	425
7	TPRG1	425
8	SPATA4	420
9	SPEF2	420
10	NXNL2	420

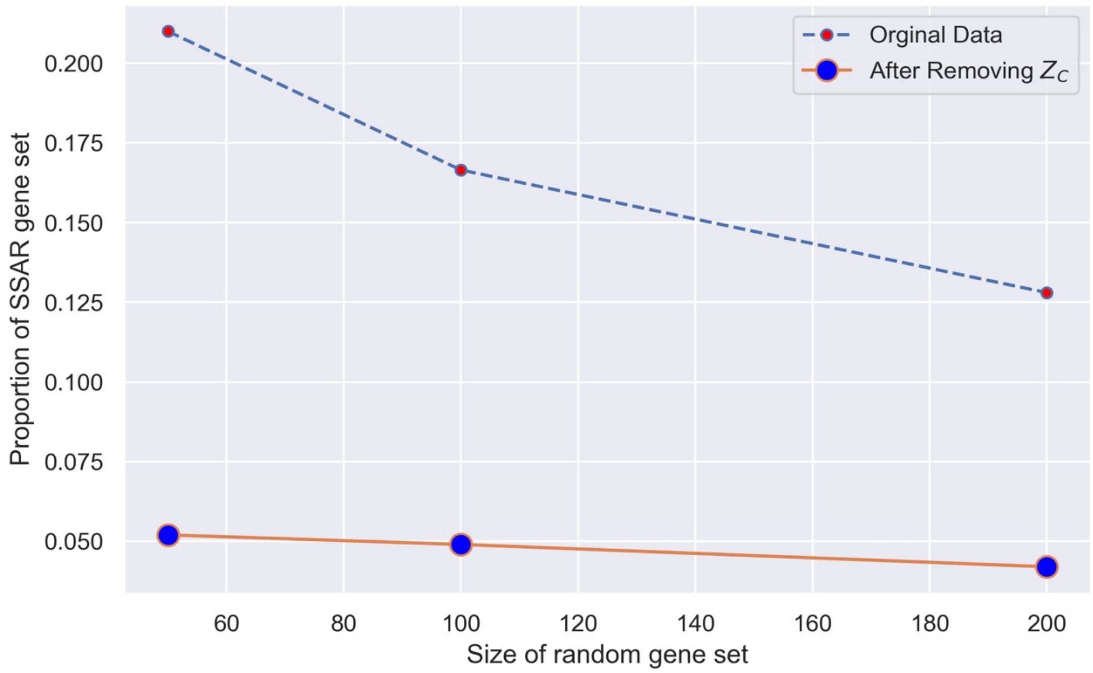

Supplement: Supplementary file 7 — Supplementary Information 4. [file 41598_2023_35588_MOESM7_ESM.pdf]
