# Supplementary material for: Removing the association of random gene sets and survival time in cancers with positive random bias using fixed-point gene set
Source: Sci Rep. 2023 May 29;13:8663. doi: 10.1038/s41598-023-35588-5 (PMC10226989; doi:10.1038/s41598-023-35588-5)
Supplement: Supplementary file 8 — Supplementary Table 1. [file 41598_2023_35588_MOESM8_ESM.pdf]

Cancer	gene	reference
**ACC**	STARD5<br>CD68	https://www.science.org/doi/abs/10.1126/science.7892608<br>https://www.ncbi.nlm.nih.gov/pmc/articles/PMC7103591/
**BLCA**	TBX2	https://pubmed.ncbi.nlm.nih.gov/22284968/<br>https://www.nature.com/articles/modpathol2014145
**BRCA**	C6orf97	https://www.nature.com/articles/ejhg2014219.pdf?origin=ppub
**GBMLGG**	EMP3	https://www.ncbi.nlm.nih.gov/pmc/articles/PMC8106853/
**HNSC**	ACTA1	https://www.ncbi.nlm.nih.gov/pmc/articles/PMC6687676/<br>https://www.proteinatlas.org/ENSG00000143632-ACTA1/pathology
**KIPAN**	CD8A<br>CD247	https://pubmed.ncbi.nlm.nih.gov/34491407/<br>https://www.frontiersin.org/articles/10.3389/fonc.2018.00456/full
**KIRC**	STEAP3	https://www.ncbi.nlm.nih.gov/pmc/articles/PMC8921746/
**KIRP**	GPR125	https://www.ncbi.nlm.nih.gov/pmc/articles/PMC5808184/
**LGG**	HTR5A	https://www.ncbi.nlm.nih.gov/pmc/articles/PMC7294948/
**LIHC**	ABCB4	https://www.ncbi.nlm.nih.gov/pmc/articles/PMC4219162/
**LUAD**	BUB1	https://pubmed.ncbi.nlm.nih.gov/11146226/<br>https://tcr.amegroups.com/article/view/42636/html
**LUSC**	AGR3	https://www.ncbi.nlm.nih.gov/pmc/articles/PMC6557199/
**MESO**	COL4A2	https://pubmed.ncbi.nlm.nih.gov/35328227/
**PAAD**	ANK2<br>NTF4	https://pubmed.ncbi.nlm.nih.gov/21042036/<br>https://www.cell.com/molecular-therapy-family/nucleic-acids/fulltext/S2162-2531(21)00156-6#fig2
**THYM**	SH2D1A	https://www.nature.com/articles/s41598-019-38878-z
**UCEC**	DNAH12	https://www.tmrjournals.com/public/articlePDF/20220712/36788d7933f1fe35d74e7e1f8bd9e3dc.pdf
**UVM**	HTR2B	https://pubmed.ncbi.nlm.nih.gov/31002821/<br>https://pubmed.ncbi.nlm.nih.gov/30347896/

S1 Table relevance of high scoring genes of fixed-point sets to corresponding cancer types.
