# Supplementary material for: Removing the association of random gene sets and survival time in cancers with positive random bias using fixed-point gene set
Source: Sci Rep. 2023 May 29;13:8663. doi: 10.1038/s41598-023-35588-5 (PMC10226989; doi:10.1038/s41598-023-35588-5)
Supplement: Supplementary file 9 — Supplementary Table 2. [file 41598_2023_35588_MOESM9_ESM.pdf]

Enriched pathway	Reference
**Primary immunodeficiency**	https://www.frontiersin.org/articles/10.3389/fimmu.2018.03136/full
**T cell receptor signaling pathway**	https://link.springer.com/article/10.1007/s13238-016-0367-1
**Cytokine-cytokine receptor interaction**	https://www.ncbi.nlm.nih.gov/pmc/articles/PMC5577299/
**Hematopoietic cell lineage**	https://www.ncbi.nlm.nih.gov/pmc/articles/PMC7290570/
**Th17 cell differentiation**	https://www.ncbi.nlm.nih.gov/pmc/articles/PMC8041630/
**PD-L1 expression and PD-1 checkpoint pathway in cancer**	https://pubmed.ncbi.nlm.nih.gov/28346916/
**Cell adhesion molecules**	https://www.ncbi.nlm.nih.gov/pmc/articles/PMC6896014/
**Cell cycle**	https://pubmed.ncbi.nlm.nih.gov/26804550/
**ECM-receptor interaction**	https://www.frontiersin.org/articles/10.3389/fcell.2019.00086/full
**Amoebiasis**	https://www.ncbi.nlm.nih.gov/pmc/articles/PMC6096082/
**Focal adhesion**	https://www.ncbi.nlm.nih.gov/pmc/articles/PMC6958047/
**Protein digestion and absorption**	http://www.aimspress.com/article/id/4377
**Metabolic pathways**	https://www.ncbi.nlm.nih.gov/pmc/articles/PMC4792565/
**Dilated cardiomyopathy**	https://www.ncbi.nlm.nih.gov/pmc/articles/PMC8918393/
**Pathways in cancer**	
**Chemokine signaling pathway**	https://www.ncbi.nlm.nih.gov/pmc/articles/PMC2907742/
**Metabolism of xenobiotics by cytochrome**	https://pubmed.ncbi.nlm.nih.gov/21184128/
**Viral protein interaction with cytokine and cytokine receptor**	https://www.ncbi.nlm.nih.gov/pmc/articles/PMC4074797/
**Arrhythmogenic right ventricular cardiomyopathy**	https://www.mdpi.com/2077-0383/9/6/1702/htm
**Nicotine addiction**	https://www.ncbi.nlm.nih.gov/pmc/articles/PMC3085312/
**Chemical carcinogenesis - DNA adducts**	https://www.ncbi.nlm.nih.gov/pmc/articles/PMC4439668/
**Hypertrophic cardiomyopathy**	https://www.ncbi.nlm.nih.gov/pmc/articles/PMC7356401/
**Retinol metabolism**	https://www.ncbi.nlm.nih.gov/pmc/articles/PMC2991380/
**Morphine addiction**	https://www.ncbi.nlm.nih.gov/pmc/articles/PMC3158334/
**Progesterone-mediated oocyte maturation**	https://pubmed.ncbi.nlm.nih.gov/26804550/

**Th1 and Th2 cell differentiation**	https://www.frontiersin.org/articles/10.3389/fphar.2019.00624/full
**Drug metabolism - cytochrome P450**	https://www.ncbi.nlm.nih.gov/pmc/articles/PMC6171375/
**Fc epsilon RI signaling pathway**	https://pubmed.ncbi.nlm.nih.gov/16339523/
**Natural killer cell mediated cytotoxicity**	https://molecular-cancer.biomedcentral.com/articles/10.1186/s12943-020-01238-x
**Synaptic vesicle cycle**	https://pubmed.ncbi.nlm.nih.gov/24965146/ https://www.frontiersin.org/articles/10.3389/fbioe.2020.00177/full#B30
**GABAergic synapse**	https://www.ncbi.nlm.nih.gov/pmc/articles/PMC2931807/
**Osteoclast differentiation**	https://www.nature.com/articles/s41413-020-00105-1

S2 Table Enriched pathway of fixed-point sets and their relevance to cancer.
